# Angiotensin II Reduces Lipoprotein Lipase Expression in Visceral Adipose Tissue via Phospholipase C β4 Depending on Feeding but Increases Lipoprotein Lipase Expression in Subcutaneous Adipose Tissue via c-Src

**DOI:** 10.1371/journal.pone.0139638

**Published:** 2015-10-08

**Authors:** Tsuyoshi Uchiyama, Shoichi Tomono, Koichi Sato, Tetsuya Nakamura, Masahiko Kurabayashi, Fumikazu Okajima

**Affiliations:** 1 Laboratory of Signal Transduction, Institute for Molecular and Cellular Regulation, Gunma University, Maebashi, Gunma, Japan; 2 Gunma University School of Health Sciences, Maebashi, Gunma, Japan; 3 Clinical Investigation Unit, Gunma University, Maebashi, Gunma, Japan; 4 Department of Medicine and Biological Science, Gunma University, Maebashi, Gunma, Japan; Tohoku University, JAPAN

## Abstract

Metabolic syndrome is characterized by visceral adiposity, insulin resistance, high triglyceride (TG)- and low high-density lipoprotein cholesterol-levels, hypertension, and diabetes—all of which often cause cardiovascular and cerebrovascular diseases. It remains unclear, however, why visceral adiposity but not subcutaneous adiposity causes insulin resistance and other pathological situations. Lipoprotein lipase (LPL) catalyzes hydrolysis of TG in plasma lipoproteins. In the present study, we investigated whether the effects of angiotensin II (AngII) on TG metabolism are mediated through an effect on LPL expression. Adipose tissues were divided into visceral adipose tissue (VAT) and subcutaneous adipose tissue (SAT) for comparison. AngII accelerated LPL expression in SAT but, on the contrary, suppressed its expression in VAT. In both SAT and VAT, AngII signaled through the same type 1 receptor. In SAT, AngII increased LPL expression via c-Src and p38 MAPK signaling. In VAT, however, AngII reduced LPL expression via the G_q_ class of G proteins and the subsequent phospholipase C β4 (PLCβ4), protein kinase C β1, nuclear factor κB, and inducible nitric oxide synthase signaling pathways. PLCβ4 small interfering RNA experiments showed that PLCβ4 expression is important for the AngII-induced LPL reduction in VAT, in which PLCβ4 expression increases in the evening and falls at night. Interestingly, PLCβ4 expression in VAT decreased with fasting, while AngII did not decrease LPL expression in VAT in a fasting state. In conclusion, AngII reduces LPL expression through PLCβ4, the expression of which is regulated by feeding in VAT, whereas AngII increases LPL expression in SAT. The different effects of AngII on LPL expression and, hence, TG metabolism in VAT and SAT may partly explain their different contributions to the development of metabolic syndrome.

## Introduction

The triglyceride (TG) lipase gene subfamily is comprised of three evolutionarily related lipases, i.e., lipoprotein lipase (LPL), hepatic lipase, and endothelial lipase, and plays a central role in plasma lipoprotein metabolism and homeostasis. These lipases are differentiated by their tissue-specific expression and substrate specificity [1,2]. LPL is a central enzyme in overall TG metabolism and plays a crucial role in lipid homeostasis and energy balance. The LPL that is mainly synthesized within muscle cells, cardiomyocytes, and adipocytes migrates to the vascular endothelium surface, where TG in very low-density lipoprotein and chylomicron is hydrolyzed to glycerol and fatty acids, and these products are taken inside the cells [2].

Adipocytes are distributed over the entire body and are classified into white and brown adipose tissues. In some humans, fat in white adipose tissues increases, especially in the abdomen, with age, sometimes resulting in a cluster of pathological conditions that is called metabolic syndrome. White adipose tissues are divided into subcutaneous and visceral adipose tissues depending on their localization [3]. Metabolic syndrome is characterized by visceral adiposity, insulin resistance, dyslipidemia, hypertension, and diabetes [4–6]. These pathological conditions often cause cardiovascular and cerebrovascular diseases. Many epidemiological studies support the notion that visceral adiposity increases the risk of disorders, such as diabetes, hypertension, hypertriglyceridemia, and atherosclerosis [4,5]. For example, a recent study using 1511 individuals in the MESA (Multi-Ethnic Study of Atherosclerosis) with adiposity assessment by computed tomography (CT) suggested that visceral adiposity is essential to assessing cardiometabolic risk, regardless of age, race, and body mass index [7]. It is not fully understood, however, why visceral adipose tissue (VAT) but not subcutaneous adipose tissue (SAT) brings about insulin resistance and related events [4–7].

Hypertension, one diagnostic criterion of metabolic syndrome, is regulated by the renin-angiotensin system [8] and angiotensin II (AngII) is important as a target of antihypertensive drugs. Although the major source of circulating angiotensinogen is liver, recent studies have shown that the renin-angiotensin system is working in adipocytes and regulates their functions [9,10]. For example, in angiotensinogen-knockout mice, fat levels are decreased, which shows that angiotensin is important for adipocyte differentiation [11]. Similarly, mice lacking angiotensin-converting enzyme had lower body weight and a lower proportion of body fat, especially in the abdomen, which was associated with increases in LPL expression [12]. In clinical studies, the secretion of angiotensin from adipose tissues has been shown to be elevated in obesity [13]. It has been reported that low LPL reflects insulin resistance and that LPL expression increased in diabetic patients with an average body mass index of 25.1 (the Japanese obesity criteria) with angiotensin receptor type 1 (ATR1) blocker treatment [14]. Moreover, in obese subjects with type 2 diabetes mellitus, circulating AngII levels correlate with changes in body weight and tend to correlate negatively with change in LPL [15]. In vitro, a long time exposure to ATR1 blockers leads to the differentiation of 3T3L-1 cells to adipocytes and induces LPL expression [16]. Thus, in vitro and in vivo observations suggest that the renin-angiotensin system regulates differentiation, growth, and LPL expression of adipocytes. However, the regulatory role and molecular mechanism of AngII in LPL expression in different types of white adipose tissues remain unknown. In the present study, we hypothesized that the difference in the AngII regulation of LPL metabolism in either VAT or SAT may explain the difference in their contributions to hypertriglyceridemia, a component of metabolic syndrome. To this end, we investigated the effects and mechanisms of AngII in regulating the expression of LPL in either VAT or SAT and the respective adipocytes prepared from adult rats. Our results indicated that AngII inhibits LPL expression in VAT, on the contrary, it stimulates LPL expression in SAT through the same ATR1 but through different intracellular signaling pathways.

## Material and Methods

### Ethics Statement

This study was carried out in strict accordance with the guidelines of the Animal Care and Experimentation Committee of Gunma University. All experimental procedures were performed in accordance with the guidelines of the animal care and experimentation committee at Gunma University. The protocol was approved by the Animal Care and Experimentation Committee of Gunma University (Permit Number: 14–29). Rats were sacrificed using diethyl ether and all efforts were made to minimize suffering.

### Animals

Male Wister rats, 8–12 weeks old (body weight: 300 g- 350 g), were purchased from Charles River Laboratory Japan (Yokohama, Japan). Rats were bred in a breeding room approved by the university with a lighting cycle of 12 h light (7 a.m. to 7 p.m.) / 12 h dark (7 p.m. to 7 a.m.). The rats were allowed to feed and drink water freely. For daily changes of the responses, the rats were maintained under the same conditions of lighting and dark cycle and allowed to feed and drink water freely, except for the starvation experiments, in which diet was removed from 1 a.m. to 1 p.m. (12 h starvation).

### Materials

DMEM, fetal bovine serum, calf serum, collagenase type I, Opti-MEM, Lipofectamine-RNAiMAX, and Stealth siRNA (PLCβ4: PLCb4RSS–332540, Scrambled: Negative control GC Duplex#2) were purchased from Invitrogen (Grand Island, NY); insulin was from the Cell Science and Technology Institute, Inc., (Miyagi Japan); antibody against LPL was from OriGene Technologies, Inc., (Rockville, MD); antibody against phospho-PKCβ1 was from Santa Cruz Biotechnology Inc., (Santa Cruz, CA); antibodies against PLCβ4 and non-phospho-PKCβ1 were from R&D Systems, Inc., (Minneapolis, MN); antibodies against c-Src and IκB (32/36) were from Cell Signaling (Beverly, MA); antibody against β-actin was from Gene Tex, Inc., (Irvine, CA); angiotensin II, adenosine, DMEM medium, TRI REAGENT, protease inhibitor, and phosphatase inhibitor I and II were from Sigma Aldrich (St. Louis, MO); pertussis toxin (PTX) was from List Biological Laboratories Inc., (Campbell, CA); brilliant syber green was from Agilent technology (Santa Clara CA); an LPL activity kit was from Roar Biomedical, Inc. (New York, NY); and ECL chemiluminescence detection system was from ECL prime (GE healthcare Pittsburgh PA). YM25490 was a gift from Dr. H Taniguchi of Astellas (Tsukuba, Japan). The sources of all other reagents were the same as described previously [17]. In the present study, we used many inhibitors and activators of intracellular signaling pathways. For convenience, their action sites are summarized in S1 Table.

### Adipose tissue isolation and its culture

Rats were sacrificed using diethyl ether. The skin was peeled off from the abdominal muscle, and SAT was collected from the inguinal region. VAT was collected from the peri-renal adipose tissue except for the upper part of the kidney. Each collected adipose tissue was washed with HBSS containing 1% bovine serum albumin (BSA) (fraction V), was cut into pieces weighing approximately 100 mg, was cultured in 6 well plates in 4.5 mL DMEM supplemented with 1% FBS and 1.72 μM insulin, and then incubated at 37°C in a 5% CO_2_ incubator. The medium was changed the next day and the tissue was used for experiments another 24 h later.

### Isolation of adipocytes and fibrocytes and their culture

Adipose tissue was minced using a pair of scissors and was strongly shaken at 37°C in a water bath for 1 h with 1.3 mg/mL of collagenase type I for SAT, and 1 mg/mL of collagenase type I for VAT. The tissue was then filtered using a mesh of 320 μm pore size. For preparation of adipocytes, the filtrate was washed three times with HBSS containing 1% BSA (fraction V) without calcium and was finally washed with the adipocyte culture medium composed of DMEM containing 1000 mg/dL glucose, 3% BSA (fraction V), 15 mM HEPES, 1.72 μM insulin, 5 μM adenosine, and 100 μM β-mercaptoethanol. The adipocytes in the upper layer were then cultured in a 50 mL conical tube using 20 mL culture medium per 5 mL of packed adipocytes, and incubated at 37°C in a 5% CO_2_ incubator overnight. The next day, packed 200 μL of adipocytes were placed into a 2 mL tube and cultured with 1 mL of adipocyte culture medium at 37°C in a 5% CO_2_ incubator for 24 h [18]. As for fibrocytes, the filtrate of cell suspension prepared with collagenase treatment as described above was centrifuged at 100 rpm for 10 min, and a lower layer was collected. The lower layer was washed twice with HBSS by centrifugation (1000 rpm for 10 min at 4°C). The cells were then cultured in DMEM containing 1000 mg/dL glucose and 10% calf serum, and incubated at 37°C in a 5% CO_2_ incubator. The culture medium was changed every other day. The fibrocytes were subcultured in a 6-well plate. We used second subcultured fibrocyte in the present experiment.

### LPL activity of adipose tissue and adipocyte

For extraction of LPL, adipose tissue (approximately 100 mg) or packed adipocytes (200 μL volume containing approximately 4 x 10^6^ cells) were incubated in the adipocyte culture medium in a final volume of 3 mL for 24 h. After once washed with PBS, the tissue and adipocytes were incubated with 10 units/mL of heparin in PBS for 1 h in a 5% CO_2_ incubator and were centrifuged. The supernatant or extract was used to evaluate the activity of LPL over 1 h, based on the fluorescence change, using the LPL activity kit (Roar Biomedical, Inc. New York, NY) according to the manufacturer’s protocol. The activity of LPL of extract was measured with plate reader 2300 Enspire (Perkin Elmer Waltham, MA) with 370 nm excitation / 450 nm emission at 37°C and expressed as LPL activity per proteins in the tissues or cells. The remaining tissues or cells in the precipitate or upper layer were collected and the protein content was measured by a Pierce bicinchoninic acid (BCA) protein kit (Pierce Rockford, IL) as described previously [17].

### Protein extraction and western blot analysis

After washing adipose tissue (approximately 100 mg) or adipocytes (200 μL volume) with PBS, the tissues or cells were homogenized in lysis buffer (60 mM Tris/HCl pH 6.8, 1% TritonX, 10% glycerol, 4 mM EDTA, 3 mM DTT, protease inhibitors, and phosphatase inhibitor I and II) on ice and then centrifuged twice to remove lipids and debris. The supernatant was used as the protein extract and the protein content was measured. The proteins were separated by electrophoresis on a SDS-polyacrylamide gel and then transferred onto a nitrocellulose membrane (Bio Rad Hercules, CA). The membranes were incubated with a 1:1,000 dilution of antibodies in 5% skim milk TBS at 4°C overnight and were visualized with a 1:10,000 dilution horseradish peroxidase-linked IgG secondary antibody at room temperature for 2 h. The complexes were detected using the ECL chemiluminescence detection system and densitometry ratio was calculated based on densitometric quantification of the bands as described previously [17].

### RNA extraction and quantitative PCR

Total RNA of adipose tissue (approximately 100 mg) or adipocytes (200 μL volume) was isolated with 1 mL of TRI REAGENT (Sigma-Aldrich St. Louis, MO) and were centrifuged to remove lipid and insoluble substances according to the manufacturer’s protocol. After DNase I (Promega Madison, UI) treatment to remove possible traces of genomic DNA contaminating in the RNA preparations, 1.5 μg of total RNA was reverse transcribed using random priming and Multiscribe reverse transcriptase according to the instructions from the manufacturer (Applied Biosystems South San Francisco CA). The cDNA was diluted to 50 ng, 25 ng and 12.5 ng per tube, and were used for real time quantitative PCR analysis using Brilliant II SYBR green QPCR Master Mix (Agilent Technologies Santa Clara CA) with Mx3000P (Agilent Technologies Santa Clara CA). The PCR conditions for analysis of the expression of LPL and β-actin were: 40 cycles of 94°C for 25 sec, 60°C for 25 sec and 72°C for 100 sec. For analysis of the expression of other proteins, PCR conditions were: 45 cycles of 94°C for 25 sec, 60°C for 40 sec, and 72°C for 100 sec. The specific primers used for PCR are described in S2 Table.

### Transfection of small interfering RNA into adipocytes

The transfection of small interfering RNA (siRNA) into adipocytes was performed by using both the lipofectin (Lipofectamine-RNAiMAX, Invitrogen Grand Island, NY) method and the electroporation method at the same time. Stealth/siRNA at a dosage of 4 times as large as that recommended by the manufacture was used (400 nM). The siRNA was mixed with Opti-MEM to achieve a volume equal to that of the packed adipocytes. The siRNA solution and adipocytes were incubated in a 50 mL conical tube for 2 h with gentle mixing every 15 min. Electroporation of siRNA was subsequently performed using Bio Rad Gene Pulsar II (Bio Rad Hercules, CA) under conditions of 4 mm gap, 400 V, and 1000Ω, twice, with mixing of the adipocytes/siRNA suspension in the interval between the electroporation, and the mixture was then incubated at 37°C in a 5% CO_2_ incubator overnight without changing the culture medium. The following day, lipid from broken adipocytes was removed and the adipocyte suspension was divided; 200 μL was placed in a 2 mL tube (for RNA extraction) to which 1 mL of adipocytes culture medium was added, and 500 μL was placed in a 15 mL conical tube (for protein extraction).

### Data analysis

Western blotting was performed at least 3 times with single or duplicate samples. All other experiments were performed in duplicate and repeated 3 times, unless otherwise stated. Data are presented as means ± SEM of at least three separate experiments. The significance of intergroup differences was determined by ANOVA. Values of *p*<0.05 were considered to indicate a significant difference.

## Results

### Increased LPL expression and activity in SAT and reduced LPL expression and activity in VAT

**Fig 1:**
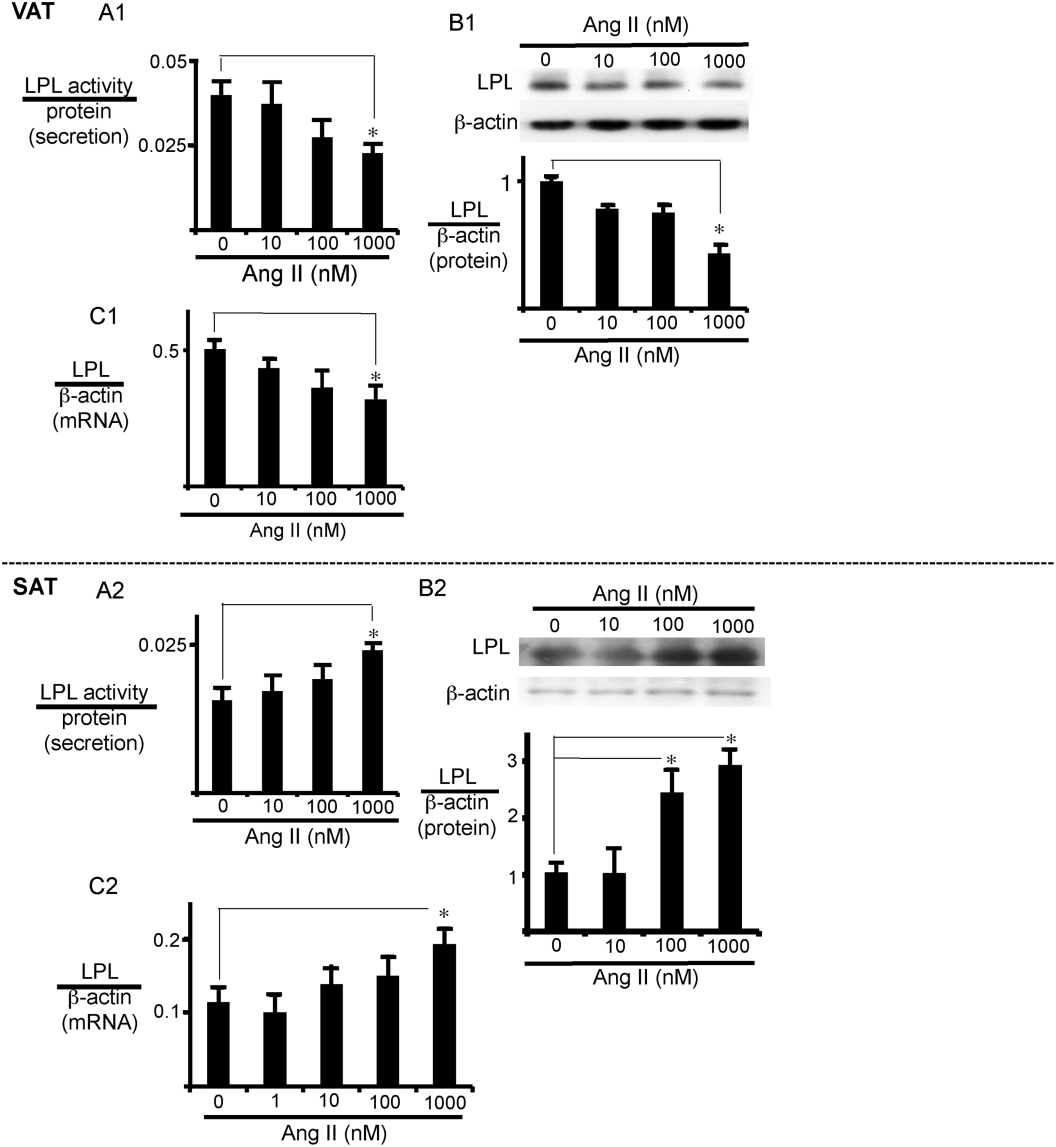
AngII has opposite effects on LPL expression in VAT and SAT. AngII dose-response of LPL expression in adipose tissues. VAT (A1, B1, and C1) or SAT (A2, B2, and C2) was incubated with AngII (vehicle, 10, 100, or 1000 nM) for 24 h and secreted LPL activity (A), LPL protein expression (B), and LPL mRNA expression (C), respectively, were measured as described in Materials and Methods. Each column and bar represents the mean ± SEM for three separate experiments. An asterisk (*) indicates *p*<0.05 vs. without AngII. The LPL activity levels were normalized with total protein, and expression levels of LPL protein and mRNA were normalized to those of β-actin.

We first examined whether AngII modifies LPL expression in SAT and VAT. In isolated VAT, AngII decreased secreted LPL activity (Fig 1A1), as well as cellular LPL protein (Fig 1B1) and mRNA expression (Fig 1C1), in a dose-dependent manner. Both the protein and mRNA expression of LPL and the activity of secreted LPL were significantly decreased by 1 μM AngII. In time course experiments, significant effects were observed 12 to 24 h after treatment with AngII (S1A1 and S1B1 Fig). In contrast, in SAT, AngII increased secreted LPL activity, as well as cellular LPL protein and mRNA expression, with significant effect at 1 μM (Fig 1A2, 1B2 and 1C2). In this case again, 24 h incubation was required for the significant effect (S1A2 and S1B2 Fig). We next examined whether modification of the expression and secretion of LPL by AngII depends on adipocytes or fibrocytes in adipose tissues. In isolated visceral adipocytes, AngII decreased secreted LPL activity (S2A1 Fig) and cellular LPL mRNA expression (S2B1 Fig). In adipocytes isolated from SAT, however, AngII increased secreted LPL activity (S2A2 Fig) and cellular LPL mRNA expression (S2B2 Fig). On the other hand, LPL mRNA expression levels in fibrocytes were much lower than those in adipocytes, and AngII did not appreciably affect LPL expression of fibrocytes of either VAT or SAT (S3A1 and S3A2 Fig). Moreover, we did not detect secreted LPL activity in fibrocytes of either VAT or SAT (data not shown).

### AngII-induced change in LPL expression mediated by ATR1 in SAT or VAT through different G proteins

**Fig 2:**
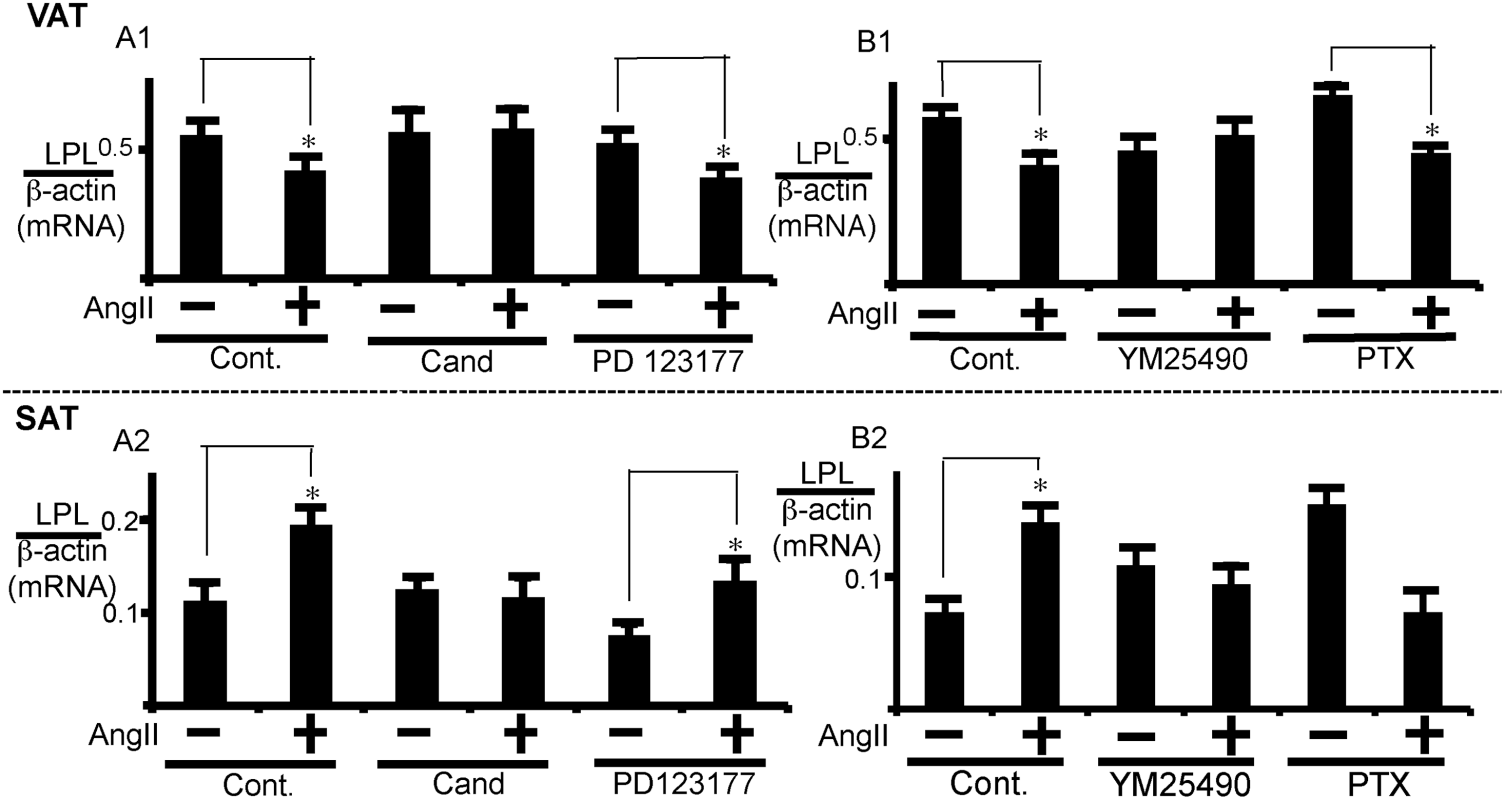
Analysis of types of AngII receptors and G proteins involved in AngII regulation of LPL expression. (A) VAT or SAT was pre-treated with 10 μM of candesartan (Cand), 5 μM of PD123177, or vehicle (Cont) for 1 h prior to AngII (1 μM) addition. (B) VAT or SAT was pre-treated with 100 nM YM25490 for 1 h or 100 ng/mL of PTX for 12 h prior to AngII (1 μM) addition. Control cells were treated with 12 h with PBS. After 24 h treatment with AngII, LPL mRNA expression was measured. Each column and bar represents the mean ± SEM for three separate experiments. An asterisk (*) indicates *p*<0.05 vs. without AngII. The LPL mRNA levels were normalized to β-actin.

We next examined whether ATR1 or angiotensin II type 2 receptor (ATR2) participates in the modulation of LPL expression by AngII. For this purpose, we examined the effects of candesartan, a selective ATR1 antagonist, and PD123177, a selective ATR2 antagonist. As shown in Fig 2A1, LPL mRNA expression was reversed by candesartan, but not by PD123177, in VAT. Similarly, candesartan, but not PD123177, was effective in attenuating AngII-induced actions in SAT (Fig 2A2). These results suggest that AngII regulates LPL expression through ATR1 in both VAT and SAT. We also examined G proteins involved in the signaling. The inhibitory effect of AngII on LPL expression was reversed by YM25490, an inhibitor of G_q/11_ proteins, but not by pertussis toxin (PTX), an inhibitor of G_i/o_ proteins, in VAT (Fig 2B1). In SAT, the stimulatory effect of AngII was also reversed by YM25490. On the other hand, PTX treatment alone increased the basal activity of LPL expression, and AngII inhibited the activity in PTX-treated cells. Thus, although we should be cautious with the PTX effects possibly due to the existence of endogenous modulators of G_i/o_ proteins, the AngII-induced stimulatory effect was not observed in the PTX-treated SAT, suggesting the coupling of G_i_ class of G proteins to ATR1 in SAT. To summarize our results, the G_q_ class of G proteins is involved in VAT, and both G_q_ and G_i_ class of G proteins may be involved in SAT in AngII-induced and ATR1-mediated signaling pathways. However, it should be noted that G protein types involved in AngII effects in SAT are not conclusive.

### Signaling pathways of AngII regulation of LPL expression in VAT and SAT

#### Involvement of phospholipase C (PLC) in VAT

The role of PLC was examined by using U73122, a PLC inhibitor, and U73343, an inactive form of U73122. In VAT, U73122, but not U73433, effectively inhibited the effect of AngII (S4A1 Fig), suggesting an involvement of PLC in the AngII-induced action in VAT. The role of PLC subtypes in VAT was extensively examined later. In SAT, however, the effects of the compounds were complex. Thus, either U73122 or U73433 inhibited AngII-induced LPL expression. Therefore, we have not yet been able to determine PLC participation in AngII-induced action in SAT (S4A2 Fig).

#### Involvement of c-Src in SAT

**Fig 3:**
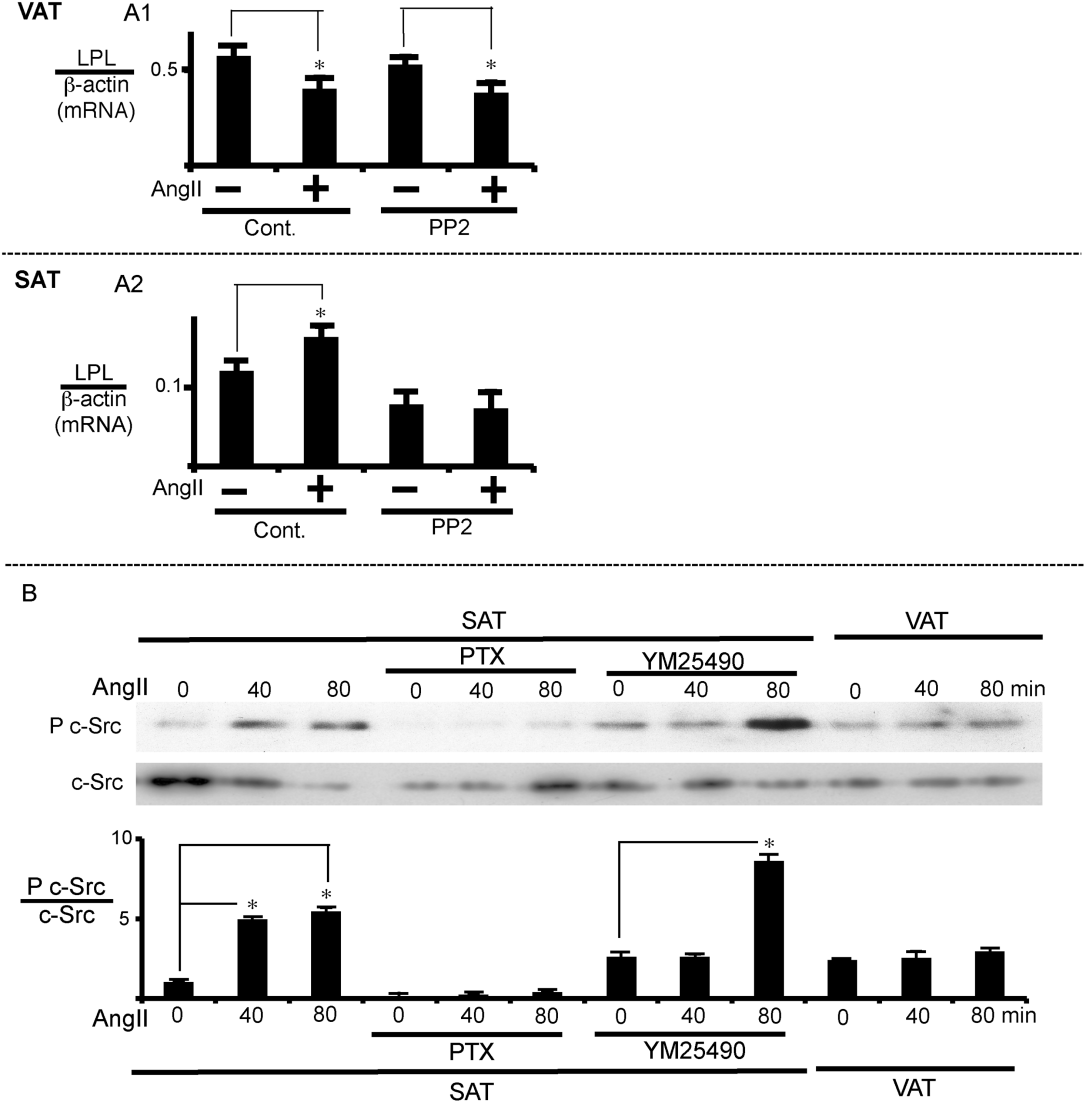
c-Src mediates the effect of AngII on LPL expression in SAT. (A) Effect of the c-Src inhibitor, PP2, on LPL expression. VAT or SAT was pre-treated with PP2 (20 μM) for 1 h prior to AngII (1 μM) addition, and incubated for 24 h to measure LPL mRNA expression. LPL mRNA levels were normalized to β-actin. (B) c-Src phosphorylation reflecting activation by AngII. SAT was pre-treated with either PTX (100 ng/mL) or YM25490 (100 nM) prior to AngII (1 μM) addition as described in Fig 2. After 24 h of AngII treatment, total protein was extracted for western blot analysis. The ratio of phospho-c-Src to total c-Src was calculated based on densitometric quantification of the bands. Each column and bar represents the mean ± SEM for three separate experiments. An asterisk (*) indicates *p*<0.05 vs. without AngII or time 0.

We next examined whether c-Src participates in the AngII-induced regulation of LPL mRNA expression. PP2, a Src-family inhibitor, reversed the AngII-induced regulation of LPL expression in SAT (Fig 3A2) but not in VAT (Fig 3A1). Consistent with the inhibitor experiment, the AngII-induced increase in LPL expression was associated with the stimulation of c-Src phosphorylation, reflecting its activation in SAT but not in VAT (Fig 3B). We also examined the effects of PTX and YM25490 on c-Src phosphorylation. PTX treatment completely inhibited AngII-induced c-Src activation (Fig 3B). The effect of YM25490 was not simple; it inhibited AngII-induced action at an earlier time point (40 min) but not a later time point of 80 min. These results suggest that AngII increased LPL expression in SAT via mainly the G_i_ class of G proteins and the subsequent c-Src activation, although involvement of the G_q_ class of G proteins cannot be ruled out.

#### Involvement of protein kinase C β1 (PKCβ1) in VAT

**Fig 4:**
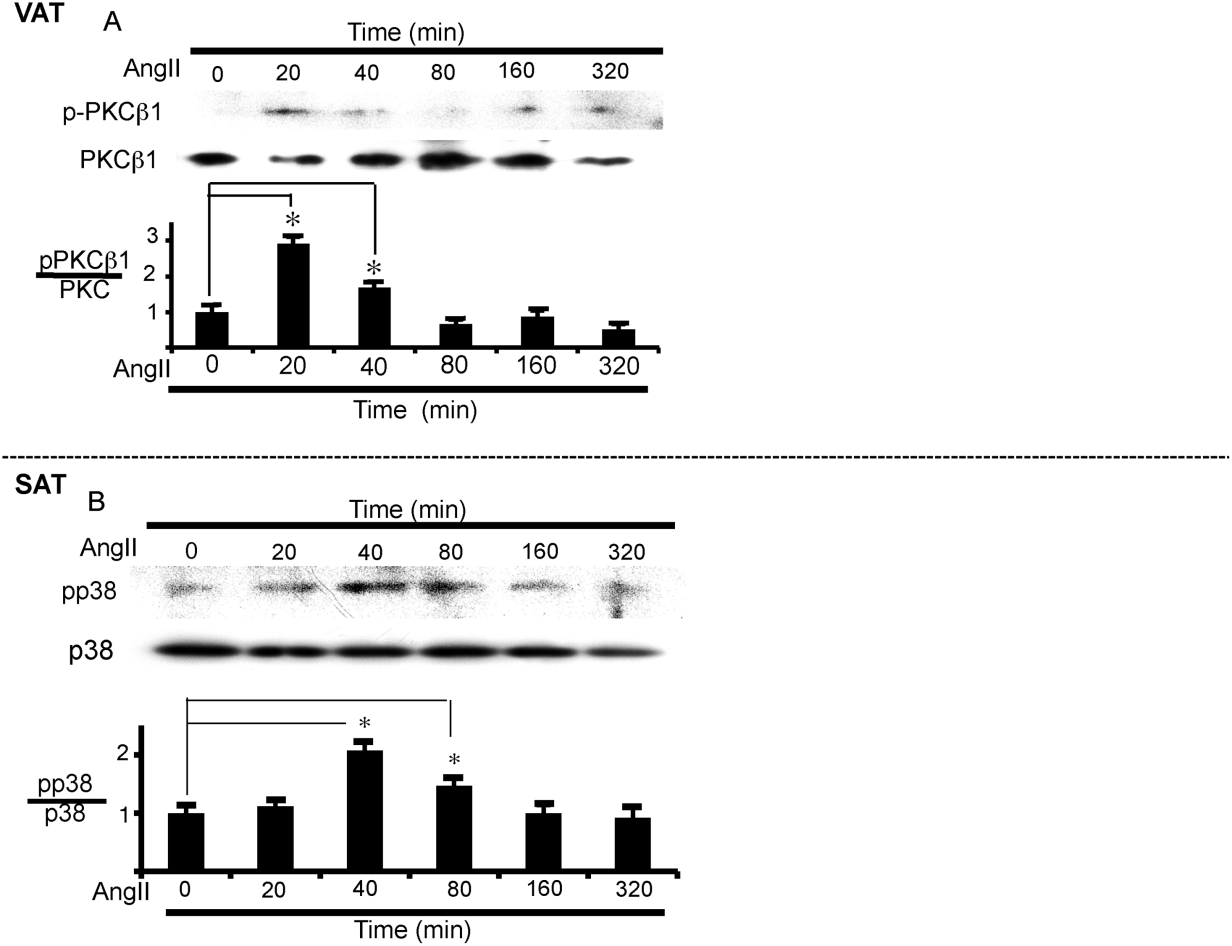
PKCβ1 and p38 MAP kinase activation by AngII in VAT and SAT, respectively. (A) PKCβ1 activation over time in VAT. VAT was incubated with AngII (1 μM) for the indicated times to measure PKCβ1 phosphorylation. The ratio of phospho-PKCβ1 to total PKCβ1 was calculated based on densitometric quantification of the bands. (B) p38 MAP kinase activation over time in SAT. SAT was incubated with AngII (1 μM) for the indicated times to measure p38MAP kinase. The ratio of phospho-p38 to total p38 was calculated based on densitometric quantification of the bands. Each column and bar represents the mean ± SEM for three separate experiments. An asterisk (*) indicates *p*<0.05 vs. time 0.

We further examined the signal transduction pathway of AngII in adipose tissues using specific inhibitors. In VAT, a non-selective PKC inhibitor, calphostin C (Cal), and PKCβ1-selective inhibitors, i.e., GO 6976 and enzastaurin, suppressed the reduction of LPL mRNA expression induced by AngII (S5A1 and S5B1 Fig). On the other hand, these inhibitors failed to inhibit the AngII-induced action in SAT (S5A2 and S5B2 Fig). Consistent with the results of the reversal of the AngII-induced LPL expression by PKCβ1 inhibitors, AngII stimulated phosphorylation of PKCβ1, reflecting activation of the enzyme, in VAT (Fig 4A). As expected from the results of Fig 2B1, AngII-induced activation of PKCβ1 was inhibited by YM25490 but not by PTX, suggesting the mediation of the G_q_ class of G proteins in PKCβ1 activation (S6 Fig).

#### Involvement of p38 MAPK in SAT

We also examined MAP kinase inhibitors. Both SB239063, a selective inhibitor of p38 MAPK, and U0126, a selective inhibitor of ERK kinase, failed to affect the AngII-induced action in VAT (S5B1 Fig); however, SB239063 inhibited the AngII-induced stimulation of LPL expression in SAT (S5B2 Fig). Supporting the effect of the p38 MAPK inhibitor, AngII induced phosphorylation of p38 MAPK in SAT, reflecting the activation of the enzyme (Fig 4B).

#### Involvement of nuclear factor κB (NFκB) and inducible nitric oxide synthase (iNOS) in VAT

**Fig 5:**
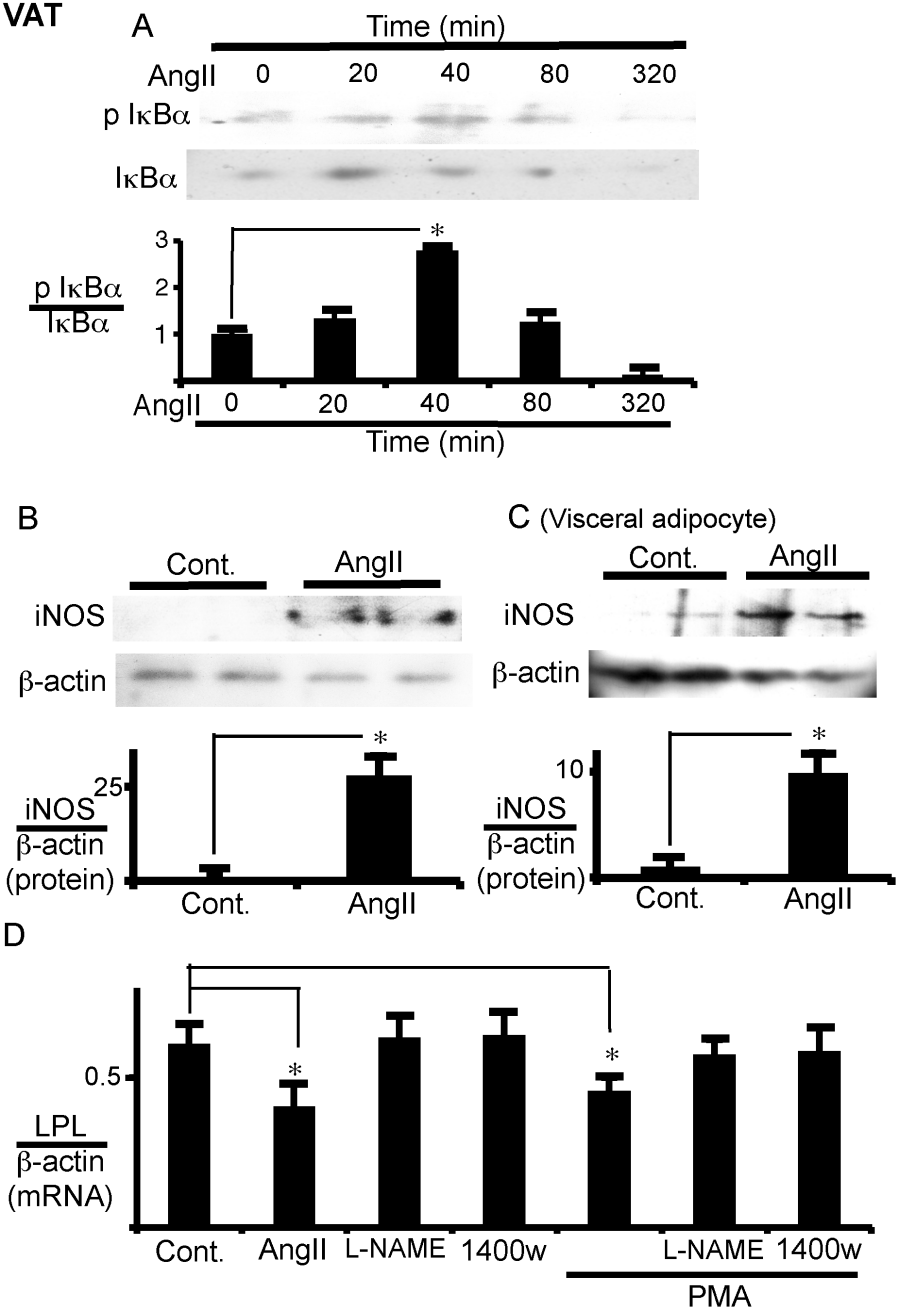
PKC, NFκB, and iNOS mediate the effect of AngII on LPL expression in VAT. (A) IκBα phosphorylation over time in VAT. VAT was cultured with AngII (1 μM) for the indicated times to measure IκBα phosphorylation with western blotting. The ratio of phospho IκBα to total IκBα was calculated based on densitometric quantification of the bands. (B, C) iNOS expression in VAT and visceral adipocytes. VAT and isolated visceral adipocytes were cultured with or without AngII (1 μM) for 24 h to measure iNOS expression by western blotting. Duplicate samples in each group were processed for western blotting. The ratio of iNOS to β-actin was calculated based on densitometric quantification of the bands (B, VAT; C, visceral adipocytes). (D) PKC is upstream of iNOS in LPL regulation in VAT. VAT was pre-treated with L-N^G^-nitroarginine Methyl Ester (L-NAME) (1 mM) or 1400w (10 nM) for 1 h prior to phorbol 12-myristate 13-acetate (PMA) (10 nM) addition. After 24 h of AngII (1 μM) or PMA treatment, LPL mRNA expression was measured. The mRNA levels were normalized with β-actin. Each column and bar represents the mean ± SEM for three separate experiments. An asterisk (*) indicates *p*<0.05 vs. time 0 or without AngII.

To determine whether NFκB is involved in AngII signaling pathways, the effects of manumycin A and Bay11-7082, IκB kinase (IKK) inhibitors, were examined. Although manumycin A was first identified as a selective farnesyltransferase inhibitor, the antibiotic is also recognized as a potent inhibitor of IKK [19]. Either manumycin A or Bay 11–7082 prevented the AngII-induced expression of LPL mRNA in VAT (S7A2 Fig), suggesting the involvement of NFκB signaling. Indeed, IκBα was phosphorylated by AngII in VAT, as assessed by western blotting using an anti-phospho IκBα antibody (Fig 5A). Either L-NAME, a NOS inhibitor, or 1400W, an iNOS-selective inhibitor, reversed the AngII-induced reduction in LPL expression; GSNO, an NO donor, inhibited LPL expression in VAT (S7B1 and S7C Fig), suggesting the involvement of iNOS in the signaling. Indeed, AngII induced iNOS expression in VAT (Fig 5B) and in isolated visceral adipocytes (Fig 5C). These results suggest that NFκB activation and the resulting NO reduced the expression of LPL. We then determined whether the AngII-induced iNOS expression is induced downstream of PKC activation or not. Both L-NAME and 1400W suppressed the reduction in LPL mRNA expression as induced by phorbol 12-myristate 13-acetate (PMA), which activates PKC (Fig 5D). These results suggest that iNOS may be induced downstream of PKC activation in VAT.

### The critical role of PLCβ4 in VAT

**Fig 6:**
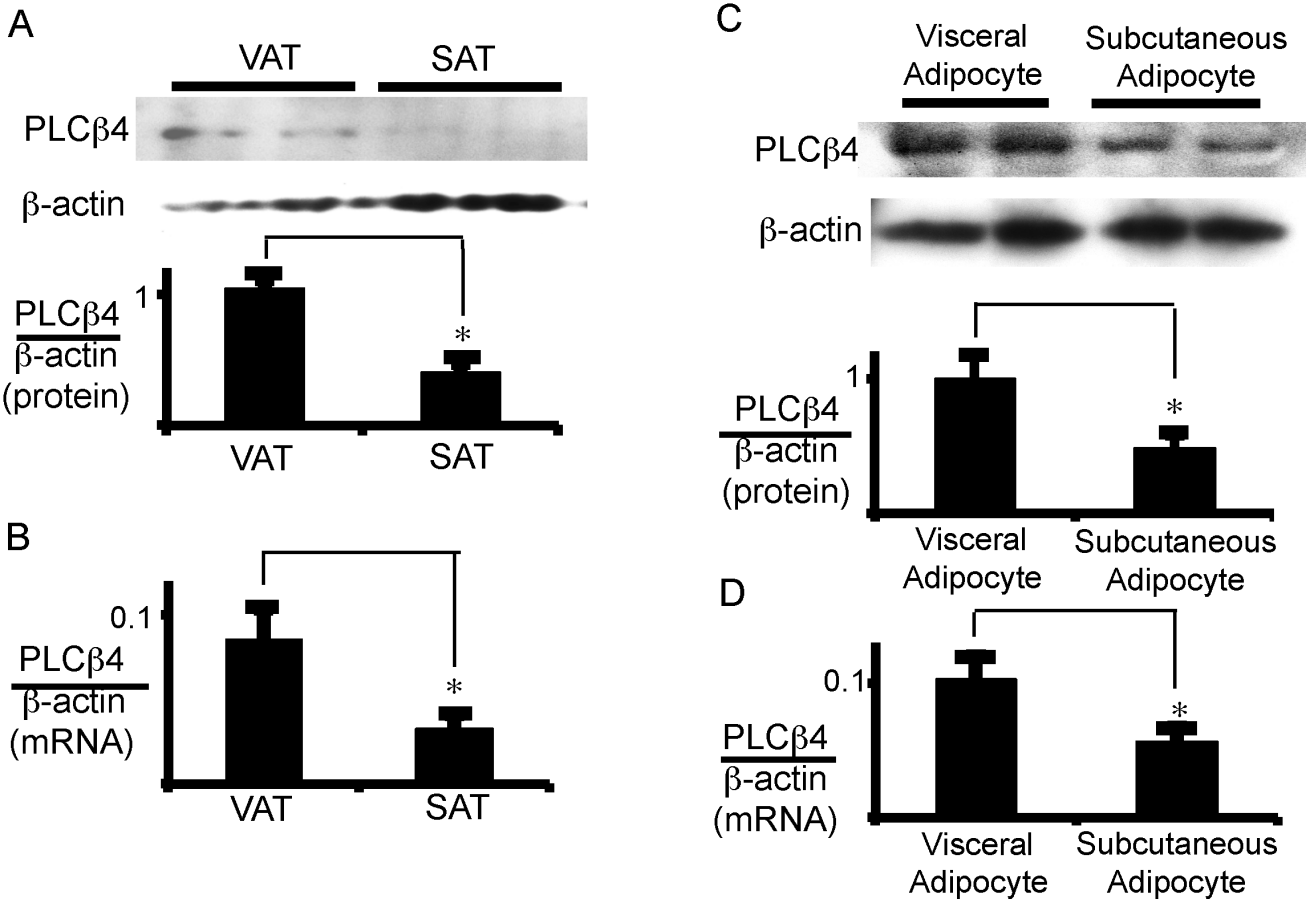
PLCβ4 is highly expressed in VAT and visceral adipocytes. Total protein (A, C) and total RNA (B, D) was extracted for western blot analysis of PLCβ4 protein and for qRT-PCR analysis of PLCβ4 mRNA, respectively (A and B, adipose tissue; C and D; adipocytes). Duplicated samples in each group were processed for western blotting. Each column and bar represents the mean ± SEM of 6 values from three separate experiments. An asterisk (*) indicates *p*<0.05 vs. VAT or visceral adipocytes. In panel A and C, the ratio of PLCβ4 to β-actin was calculated based on densitometric quantification of the bands. In panel B and D, PLCβ4 mRNA levels were normalized to β-actin.

To determine why signal transduction through the same AngII receptor differed between VAT and SAT, we examined the protein and/or mRNA critical for differentiating between VAT and SAT in AngII-induced signal transduction. The primers list employed in the present study is shown in S2 Table. There was no significant difference in the mRNA expression of the G_q_ and G_i_ classes of G proteins between SAT and VAT (S3 Table). The mRNA expression of PKCβ was significantly higher in SAT than in VAT. However, the mRNA expression of PLCβ4 was higher in VAT than in SAT (S3 Table and Fig 6B). As shown in S4 Fig, PLC seems to be involved in AngII-induced signaling; thus, we focused on PLCβ4 and further examined its expression levels in either adipose tissues or adipocytes. We confirmed its higher protein expression in VAT than in SAT (Fig 6A). Moreover, the protein and mRNA expressions of PLCβ4 in visceral adipocytes were higher than those in subcutaneous adipocytes (Fig 6C and 6D).

**Fig 7:**
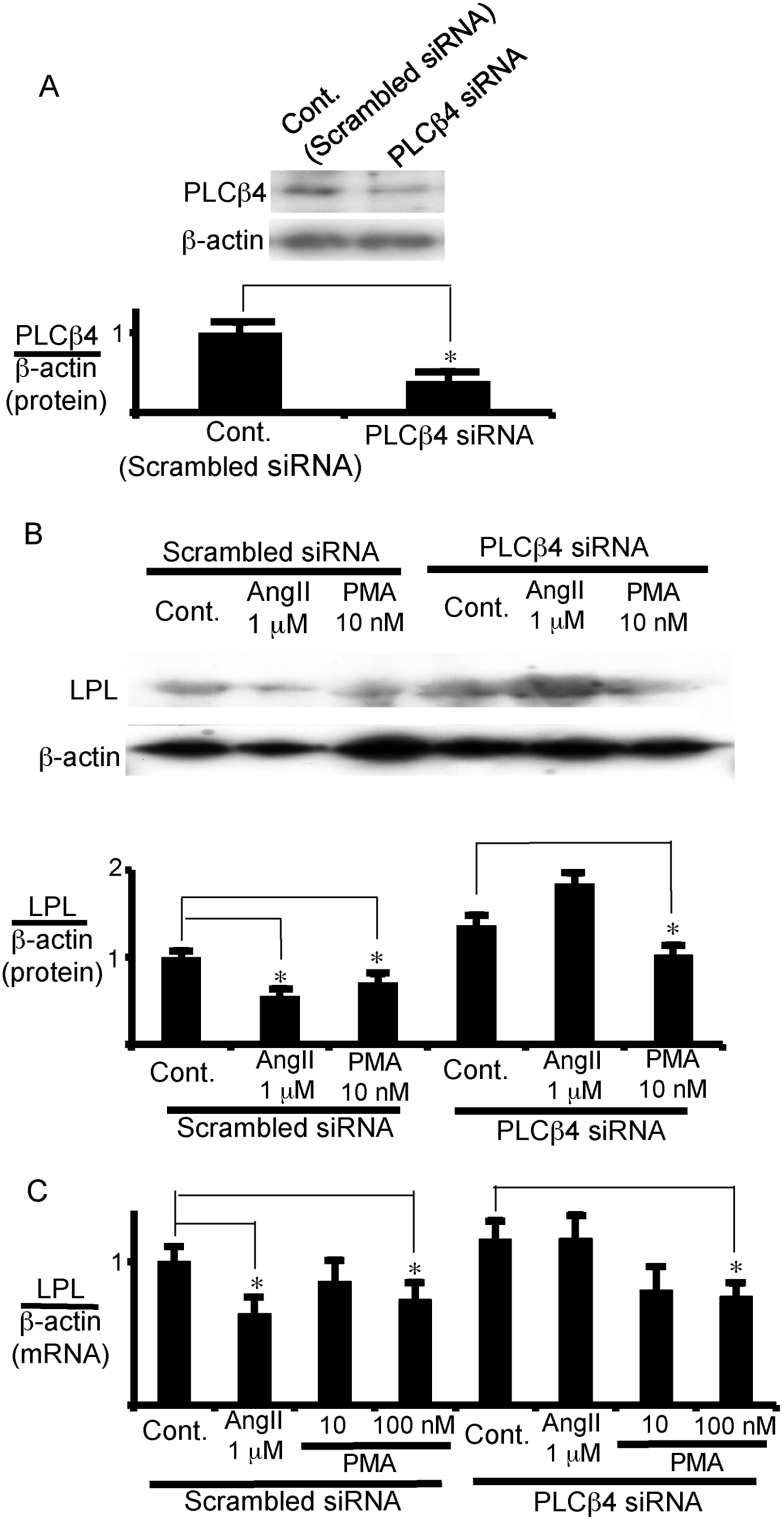
PLCβ4 is critical for the inhibition of LPL expression in visceral adipocytes. (A) siRNA specific to PLCβ4 reduced PLCβ4 expression in visceral adipocytes. Visceral adipocytes were transfected with PLCβ4-siRNA or scrambled siRNA, and incubated for 12 h to measure the enzyme expression levels by western blot analysis. (B, C) AngII reduced LPL expression via PLCβ4 in visceral adipocytes. After transfection with PLCβ4-siRNA or of scrambled siRNA, adipocytes were incubated with AngII (1 μM), PMA (10 or 100 nM), or vehicle (untreated). After 12 h incubation total protein (B) and total RNA (C) was extracted for measurement of LPL protein and mRNA levels, respectively. Each column and bar represents the mean ± SEM for three separate experiments. An asterisk (*) indicates *p*<0.05 vs. control. In panel A and B, the ratio of PLCβ4 or LPL protein to β-actin was calculated based on densitometric quantification of the bands. In panel C, LPL mRNA levels were normalized to β-actin.

To further analyze the role of PLCβ4 in the AngII regulation of LPL mRNA expression, the expression of PLCβ4 was suppressed in visceral adipocytes using small interfering RNA (siRNA) (Fig 7A). Although a third of PLCβ4 protein was still expressed by the siRNA (Fig 7A), the reduction in LPL protein and mRNA expression induced by AngII was almost completely reversed by the PLCβ4 siRNA, while this siRNA did not appreciably affect the PMA-induced suppression of LPL expression (Fig 7B and 7C). These results suggest that the AngII-induced inhibition of LPL expression is mediated by PLCβ4 and, moreover, raise the possibility that the difference in the expression level of PLCβ4 between subcutaneous and visceral adipocytes may cause the different effects of AngII on LPL expression in adipose tissues.

**Fig 8:**
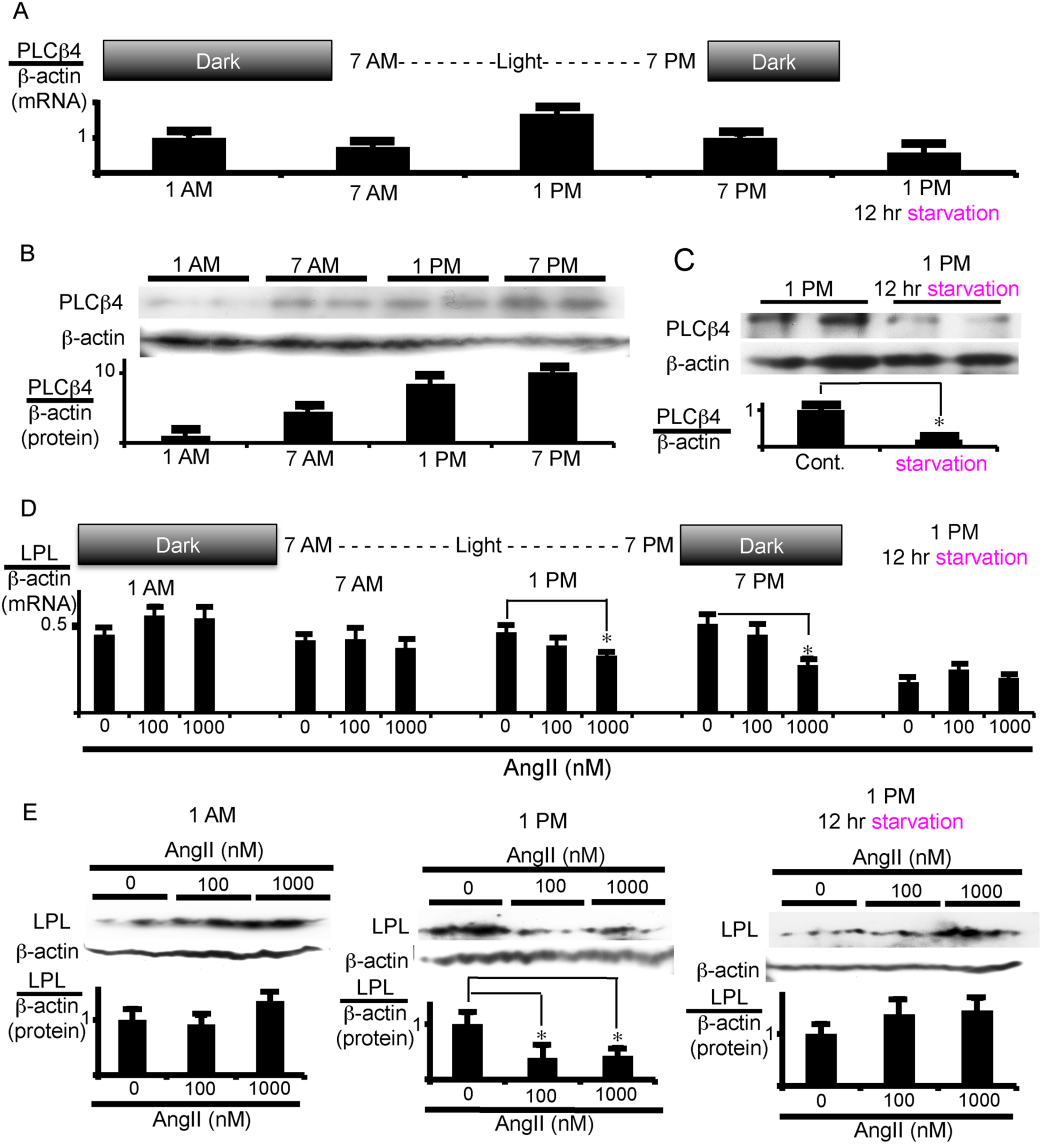
PLCβ4 expression is regulated by feeding/fasting cycle in VAT. VAT was isolated every 6 h (A, B, D, and E), or after fasting for 12 h from 1 a.m. to 1 p.m. (in the right panel of A, C, D, and E). (A-C) Daily change and starvation effects on PLCβ4 expression in VAT. Total RNA (A) or protein (B, C) was extracted for measurement of mRNA and protein expression of PLCβ4, respectively. (D, E) Effects of PLCβ4 expression levels on the LPL response to AngII in VAT. VAT was treated with the indicated doses of AngII. After 12 h incubation, LPL mRNA levels (D) and, after 18 h incubation, total LPL protein levels (E) were measured. Each column and bar represents the mean ± SEM of 4 values from 2 separate experiments for mRNA measurement (A and D) and 3 values from 3 separate experiments for western blotting experiments. An asterisk (*) indicates *p*<0.05 vs. control or without AngII. In panel B, C, and E, the ratio of LPL or PLCβ4 protein to β-actin was calculated based on densitometric quantification of the bands. In panels A and D, mRNA levels were normalized to β-actin mRNA.

It has been reported that the expression of PLCβ4 follows a circadian rhythm [20–22]. Adipose tissues and adipocytes were, therefore, taken every 6 h to examine the PLCβ4 expression in VAT. The mRNA expression of PLCβ4 was highest at 1 p.m. (Fig 8A), and its protein level was maximal from 1 to 7 p.m. (Fig 8B). Consistent with the changes in PLCβ4 expression, AngII suppressed LPL mRNA expression when added to VAT sampled at 1 p.m. or 7 p.m. but did not suppress it when VAT was sampled at 1 a.m. or 7 a.m. (Fig 8D). Similarly, AngII significantly inhibited LPL protein expression in the VAT sample collected at 1 p.m. but not at 1 a.m. (Fig 8E). Thus, AngII action seems to depend on the expression level of PLCβ4.

Finally, we examined the influence of caloric intake on PLCβ4 expression. Rats were left without food for 12 h from 1 a.m. to 1 p.m. but were allowed to freely take in water. The mRNA and protein expression of PLCβ4 in VAT significantly decreased after fasting as compared to without fasting (compare 1 p.m. with 1 p.m. 12 h starvation in Fig 8A for mRNA and in Fig 8C for protein). Moreover, the addition of AngII to starved VAT did not suppress LPL mRNA (Fig 8D, right panel) and protein expression (Fig 8E, right panel), which were totally different from the results obtained without starvation (1 p.m. in Fig 8D and 8E for mRNA and protein, respectively). These results suggest that PLCβ4 expression is regulated by feeding/fasting cycle and further support the notion that PLCβ4 levels determine whether AngII down-regulates LPL.

## Discussion

**Fig 9:**
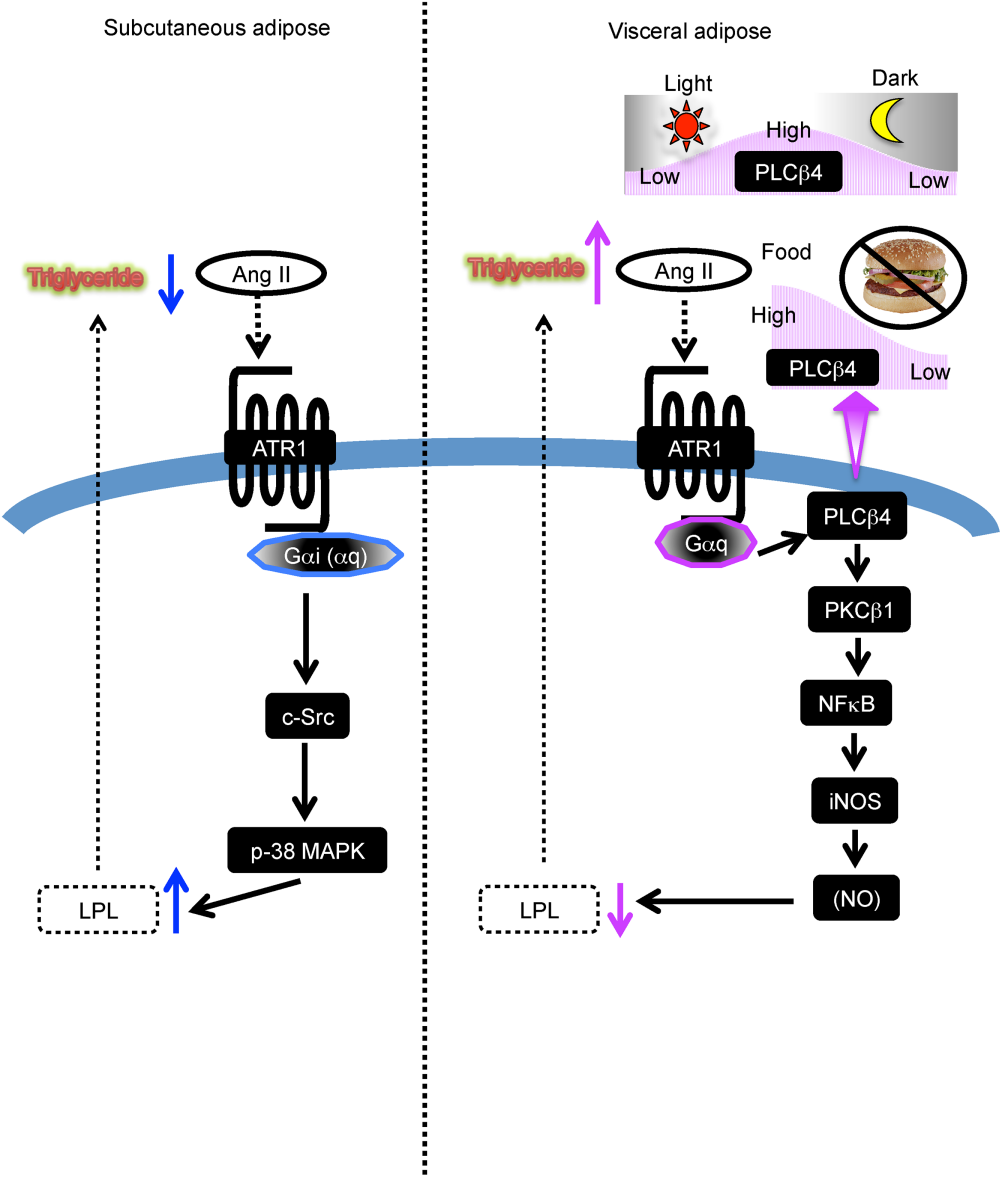
Postulated regulatory mechanism by AngII of LPL expression in VAT and SAT. AngII stimulates LPL expression in SAT and, conversely, inhibits its expression in VAT. In both cases, the AngII-induced actions are mediated by the same ATR1 but different G proteins and intracellular signaling pathways. In VAT, PLCβ4 expression is regulated by feeding/fasting cycle and is responsible for the inhibitory role of AngII on LPL expression. See text for more detail.

Metabolic syndrome is characterized by visceral adiposity, insulin resistance, hypertension, diabetes, and dyslipidemia, including hypertriglyceridemia and low plasma HDL levels; all of these conditions are closely related and often cause cardiovascular and cerebrovascular diseases [4,5,6]. A number of studies have focused on defining the differences between VAT and SAT to explain the apparent pathogenicity of visceral adiposity. It is not fully understood, however, why VAT but not SAT induces hypertriglyceridemia and its associated events leading to metabolic syndrome [4,5,6]. In the present study, we found that AngII reduced the expression of LPL, an obligatory lipase of lipoprotein-associated TG for supplying the lipolytic products to adipocytes for deposit as TG in VAT and, conversely, increased LPL expression in SAT. The inhibitory role of AngII, a well-known regulator of blood pressure, on LPL expression in VAT may partly explain the reduction of TG metabolism and resultant hypertriglyceridemia. We also examined how AngII oppositely regulates LPL expression in VAT and SAT and found a key role of PLCβ4 in the inhibition of LPL in VAT. Interestingly, PLCβ4 expression is dependent on the feeding state; levels are increased with feeding and decreased with fasting. Consequently, AngII-induced inhibition of LPL expression is also dependent on feeding. Thus, in addition to blood pressure control, AngII regulates TG metabolism, depending on the feeding/fasting cycle. The proposed mechanisms of AngII actions in either SAT or VAT are summarized in Fig 9.

As mentioned in the introduction in detail, recent studies have shown that the renin-angiotensin system is involved in the regulation of fat mass and/or LPL expression, although its different effects in VAT and SAT have not been exactly known. For example, mice lacking angiotensinogen [11] or angiotensin-converting enzyme [12] had lower body weight and a lower proportion of body fat in association with an increase in LPL expression. In obese subjects with type 2 diabetes mellitus, circulating AngII levels correlate with changes in body weight and tend to correlate negatively with change in LPL [15]. Moreover, an ATR1 antagonist, valsartan, increased blood LPL levels in type II diabetes with hypertension [14]. These clinical data suggest that the renin-angiotensin II system negatively controls LPL expression. In addition to these clinical studies, AngII has been shown to prevent in vitro differentiation of 3T3-L1 cells to adipocytes in association with the inhibition of LPL expression, which is one indicator of the adipose differentiation [16]. Therefore, the clinical data regarding the relation of the renin-angiotensin II system to LPL levels might be explained by the inhibitory role of AngII on the differentiation of adipocytes. However, the present study has shown that AngII inhibited LPL expression in isolated mature VAT and stimulated it in SAT. The inhibitory and stimulatory actions of AngII were observed in isolated adipocytes prepared from VAT and SAT, respectively, as well. In any event, the present study is the first indication that AngII can regulate LPL expression through a direct action on adipocytes in a manner independent of adipocyte differentiation, and it raises the possibility that the LPL changes observed in clinical studies may reflect the differentiation-independent action of the renin-angiotensin II system on mature adipocytes.

The physiological and pathophysiological role of AngII-induced opposite regulation of LPL in VAT and SAT is currently unknown. The LPL activity and the LPL mRNA expression level in VAT are, respectively, about 3 times and 5 times higher than those in SAT. Thus, in the basal state, the ratio of the volume of VAT and SAT may determine whether AngII increases or reduces circulating TG levels in the whole body. In the presence of AngII, the LPL activity level becomes almost the same in both adipose tissues. In the present study, we did not examine the effect of feeding on AngII-induced stimulation of LPL expression in SAT. However, we do not expect a feeding effect in SAT because of the ineffective expression level of PLCβ4, as discussed below. The inhibitory action of AngII was observed several hours after the end of feeding and continued for at least 6 h (Fig 8D). The deposition of TG in adipocytes through LPL activity after feeding may balance with lipolysis of TG in the same cells during fasting. However, inhibition by AngII of LPL expression causes the reduction of the capability of adipocytes to stock TG in VAT, resulting in the enhancement of increased plasma TG levels in the feeding state. On the other hand, in SAT, AngII increases LPL activity to deposit TG effectively in the cells to prevent hypertriglyceridemia, probably independent of the feeding/fasting cycle. The hormone effect in VAT may not be so serious under normal diet condition; the restriction of TG deposit in VAT can be overcome by the utilization of TG in other cells. However, in conditions where the diet exceeds the body’s capacity of calorie utilization and storage in the whole body, inhibitory AngII effects may cause severe hypertriglyceridemia. Although the present study does not exactly explain the pathogenicity differences of visceral adiposity and subcutaneous adiposity, it may partly explain the different abilities of VAT and SAT to metabolize circulating lipoprotein-associated TG and, hence, their ability to contribute to hypertriglyceridemia and the subsequent development of metabolic syndrome.

Although the same ATR1 mediates the regulation of LPL expression, AngII inhibited and stimulated LPL expression in VAT and in SAT, respectively. The expression levels of PLCβ4 are responsible for the opposite regulation of LPL expression in VAT and SAT. In VAT, the treatment of siRNA for PLCβ4 completely reversed the AngII-induced inhibition of LPL expression, even though roughly 30% of the protein is still expressed in siRNA-treated visceral adipocytes (Fig 7). PLCβ4 is expressed in SAT at roughly one third of the expression levels in VAT; however, no inhibitory effect was observed in SAT. Considering the results of the siRNA experiment in VAT, the low expression levels of PLCβ4 in SAT may not be enough to exert appreciable inhibitory action.

There are four isozymes of PLCβ. PLCβ4 has been shown to be expressed in neurologic tissue but also in liver cells and in the heart [22,23], and is activated by the G_q_ class of the Gα subunit but not by Gβγ subunits [24]. Here, we found that PLCβ4 is expressed in adipocytes and the enzyme is also regulated by the G_q_ class of G proteins, as indicated by the inhibition by YM25490, a specific inhibitor of G_q/11_ proteins. In SAT, the G_i_ class rather than the G_q_ class of G proteins is critical for the stimulation of LPL expression as evidenced by the inhibition of AngII-induced actions with PTX. Our results suggest that the downstream signaling pathways of the G_q_ class of G proteins/PLCβ4 are PKCβ1, NFκB, and iNOS for the inhibition of LPL expression in VAT (Fig 9). On the other hand, AngII signals through the G_i_ class of G proteins/c-Src/p38 MAPK to stimulate LPL in SAT, although the involvement of the G_q_ class of G proteins can not be excluded (Fig 9). Presently, except for PLCβ4, the differences in the expression levels of signaling molecules which are assumed to be involved in VAT and SAT are unknown. We tentatively speculate that the inhibitory signals overcome the stimulatory signals in VAT and vice versa in SAT.

PLCβ4 expression is regulated with a circadian rhythm by light and temperature in Drosophila [25,26]. PLCβ4 expression increases to its maximum level as it becomes dark [20]. In a previous article, the expression of PLCβ4 mRNA was synchronized with the protein expression of the total intracellular PLCβ4 in hepatocytes [22]. Thus it has been shown that PLCβ4 protein expression is very unstable and oscillates over a light-dark cycle. We also observed daily changes of PLCβ4 in VAT in the present study. However, the protein expression of PLCβ4 did not exactly synchronize with mRNA expression. The peak of mRNA and protein expression differed by approximately 6 h. Whether the daily change in PLCβ4 in VAT reflects the circadian rhythm is currently unknown. All we can say at present is that PLCβ4 expression is regulated by the feeding/fasting cycle. Further experiments are required to examine the assumption of the circadian rhythm of PLCβ4 expression in VAT.

## Conclusions

Despite extensive previous studies, why VAT but not SAT is linked to the development of metabolic syndrome is not fully known. LPL is a lipoprotein-associated TG-degrading enzyme. Here, we presented the results showing that AngII, a potent inducer of hypertension, also regulates TG metabolism in adipocytes; the hormone suppressed LPL expression in VAT but, conversely, enhanced it in SAT. PLCβ4, whose expression is regulated by eating habits, is critical for the inhibitory AngII effect on LPL expression in VAT. AngII regulation of TG metabolism may partly explain differences in the abilities of VAT and SAT to contribute to hypertriglyceridemia, a critical component of metabolic syndrome, in some metabolic situations.

## Supporting Information

S1 Fig Time course of AngII-induced change in LPL expression in VAT and SAT. VAT or SAT was incubated with AngII (1 μM) for 3, 6, 12, or 24 h as indicated to measure LPL protein (A) and mRNA (B) expression. Each column and bar represents the mean ± SEM for three separate experiments performed. An asterisk (*) indicates *p*<0.05 vs. time 0.
(TIFF)

S2 Fig Dose-response effects of AngII on LPL expression in visceral and subcutaneous adipocytes. Visceral or subcutaneous adipocytes were incubated with the indicated concentrations of AngII for 24 h to measure LPL activity (A) and its mRNA expression (B). Each column and bar represents the mean ± SEM for three separate experiments. An asterisk (*) indicates *p*<0.05 vs. without AngII. In panels A, the LPL activity levels were normalized with total protein. In panels B LPL mRNA levels were normalized to β-actin.
(TIFF)

S3 Fig Dose-response effects of AngII on LPL expression in visceral and subcutaneous fibrocytes. Visceral or subcutaneous fibrocytes were incubated with the indicated concentrations of AngII for 24 h to measure LPL activity. Each column and bar represents the mean ± SEM for three separate experiments. An asterisk (*) indicates *p*<0.05 vs. without AngII. mRNA levels were normalized to β-actin.
(TIFF)

S4 Fig Effects of the PLC inhibitor on LPL responses to AngII in adipose tissues. VAT or SAT were treated with U73122 (10 μM), U73343 (10 μM), or vehicle (Cont) for 1 h prior to AngII (1 μM) addition, and further incubated for 24 h to measure LPL mRNA. Each column and bar represents the mean ± SEM for three separate experiments. An asterisk (*) indicates *p*<0.05 vs. without AngII. LPL mRNA levels were normalized to β-actin.
(TIFF)

S5 Fig The effects of inhibitors of PKC, p38 MAP kinase, and ERK on LPL responses to AngII in adipose tissues. (A) VAT or SAT was treated with inhibitors of PKCβ1, i.e., 10 nM Enzastaurin (LY317615) or 10 μM GO6976, for 1 h prior to AngII (1 μM) addition, and further incubated for 24 h to measure LPL mRNA expression. (B) VAT or SAT was treated with 50 or 100 nM calphostin C (Cal), 1 or 2 μM SB239063, or 10 μM U0126 for 1 h prior to angiotensin II (1 μM) addition, and further incubated for 24 h to measure LPL mRNA expression. Each column and bar represents the mean ± SEM for three separate experiments. An asterisk (*) indicates *p*<0.05 vs. without AngII. The mRNA levels were normalized to β-actin.
(TIFF)

S6 Fig Effects of inhibitors of G proteins on AngII-induced PKC activation in VAT. VAT was pre-treated with either PTX (100 ng/mL) or YM25490 (100 nM) for 1 h prior to AngII (1 μM) addition as described in Fig 2, and incubated for the indicated times to measure the PKCβ1 phosphorylation. The ratio of p-PKCβ1 to total PKCβ1 was calculated based on densitometric quantification of the bands. Each column and bar represents the mean ± SEM for three separate experiments performed in duplicate. An asterisk (*) indicates *p*<0.05 vs. without AngII.
(TIFF)

S7 Fig Effects of modulators of IκB kinase and iNOS on LPL responses to Angii in VAT and SAT. (A) Effects of IκB kinase inhibitors. VAT (A1) or SAT (A2) was pre-treated with Manumycin A (5 μM) or Bay11-7082 (5 μM) for 1 h prior to AngII (1 μM) addition and incubated for 24 h to measure LPL mRNA expression. (B and C) Effects of NOS regulators. VAT (B1 and C) or SAT (B2) was pre-treated with L-N^G^-nitroarginine methyl ester (L-NAME, 1 mM) or an iNOS-specific inhibitor 1400w (10 nM) for 1 h prior to AngII (1 μM) addition and incubated for 24 h to measure LPL mRNA expression. Adipose tissues were similarly incubated with S-nitro-L-glutathione (GSNO, 0.5 mM). Each column and bar represents the mean ± SEM for three separate experiments. An asterisk (*) indicates *p*<0.05 vs. without AngII. The mRNA levels were normalized to β-actin.
(TIFF)

S1 Table Action sites of specific inhibitors and activators. (TIFF)

S2 Table The primer sequences used for quantitative PCR analysis of rat G protein subunits and signal transduction proteins. GRK, G protein-coupled receptor kinase; RGS, regulator of G protein signaling; PLC, phospholipase C; PKC, protein kinase C; LPL, lipoprotein lipase; SAT, subcutaneous adipose tissue; and VAT, visceral adipose tissue.
(TIFF)

S3 Table The mRNA expression of G-protein subunits and signal transduction proteins in VAT and SAT. Total RNA was extracted from each adipose tissue and expression of the indicated signaling proteins was analyzed using qRT-PCR. The primers used are shown in S2 Table. An asterisk (*) indicates *p*<0.05 vs. vehicle tissue. GRK, G protein-coupled receptor kinase; RGS, regulator of G protein signaling; PLC, phospholipase C; PKC, protein kinase C; LPL, lipoprotein lipase; SAT, subcutaneous adipose tissue; and VAT, visceral adipose tissue.
(TIFF)
