# Supplementary figures and images for: Angiotensin II Reduces Lipoprotein Lipase Expression in Visceral Adipose Tissue via Phospholipase C β4 Depending on Feeding but Increases Lipoprotein Lipase Expression in Subcutaneous Adipose Tissue via c-Src
Source: PLoS One. 2015 Oct 8;10(10):e0139638. doi: 10.1371/journal.pone.0139638 (PMC4598143; doi:10.1371/journal.pone.0139638)

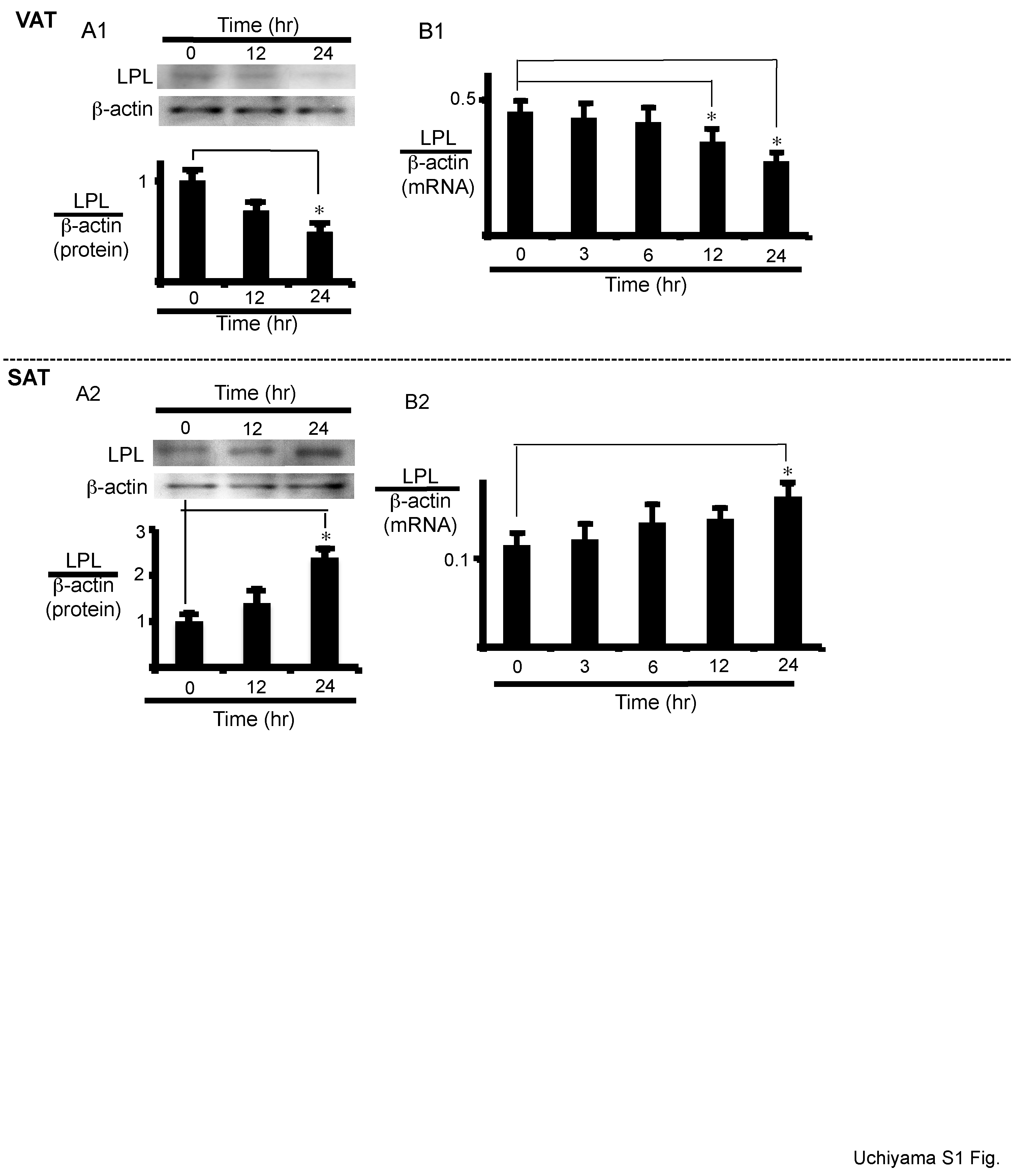

Supplement: S1 Fig — VAT or SAT was incubated with AngII (1 μM) for 3, 6, 12, or 24 h as indicated to measure LPL protein (A) and mRNA (B) expression. Each column and bar represents the mean ± SEM for three separate experiments performed. An asterisk (*) indicates p<0.05 vs. time 0. (TIFF) [file pone.0139638.s001.tiff]

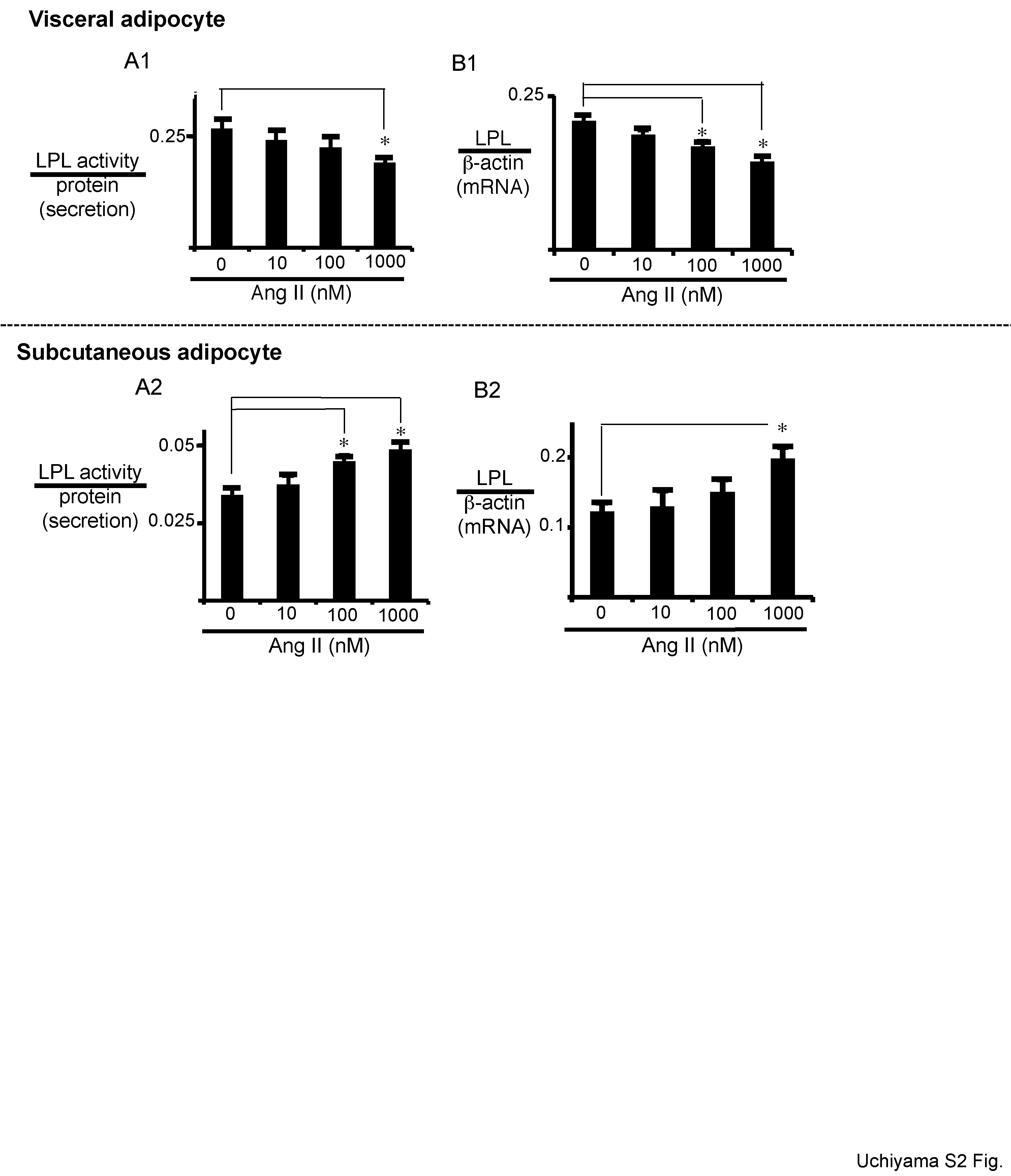

Supplement: S2 Fig — Visceral or subcutaneous adipocytes were incubated with the indicated concentrations of AngII for 24 h to measure LPL activity (A) and its mRNA expression (B). Each column and bar represents the mean ± SEM for three separate experiments. An asterisk (*) indicates p<0.05 vs. without AngII. In panels A, the LPL activity levels were normalized with total protein. In panels B LPL mRNA levels were normalized to β-actin. (TIFF) [file pone.0139638.s002.tiff]

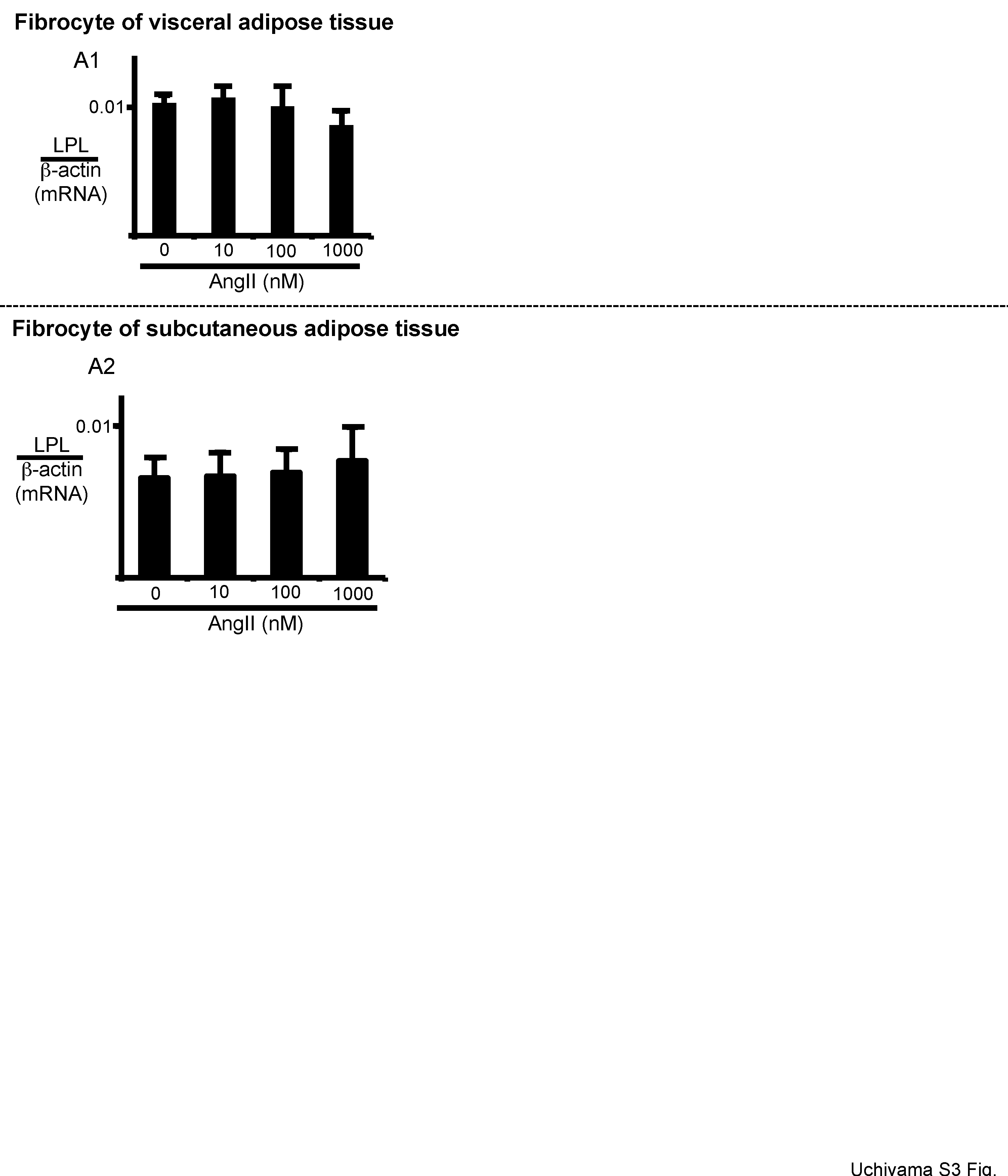

Supplement: S3 Fig — Visceral or subcutaneous fibrocytes were incubated with the indicated concentrations of AngII for 24 h to measure LPL activity. Each column and bar represents the mean ± SEM for three separate experiments. An asterisk (*) indicates p<0.05 vs. without AngII. mRNA levels were normalized to β-actin. (TIFF) [file pone.0139638.s003.tiff]

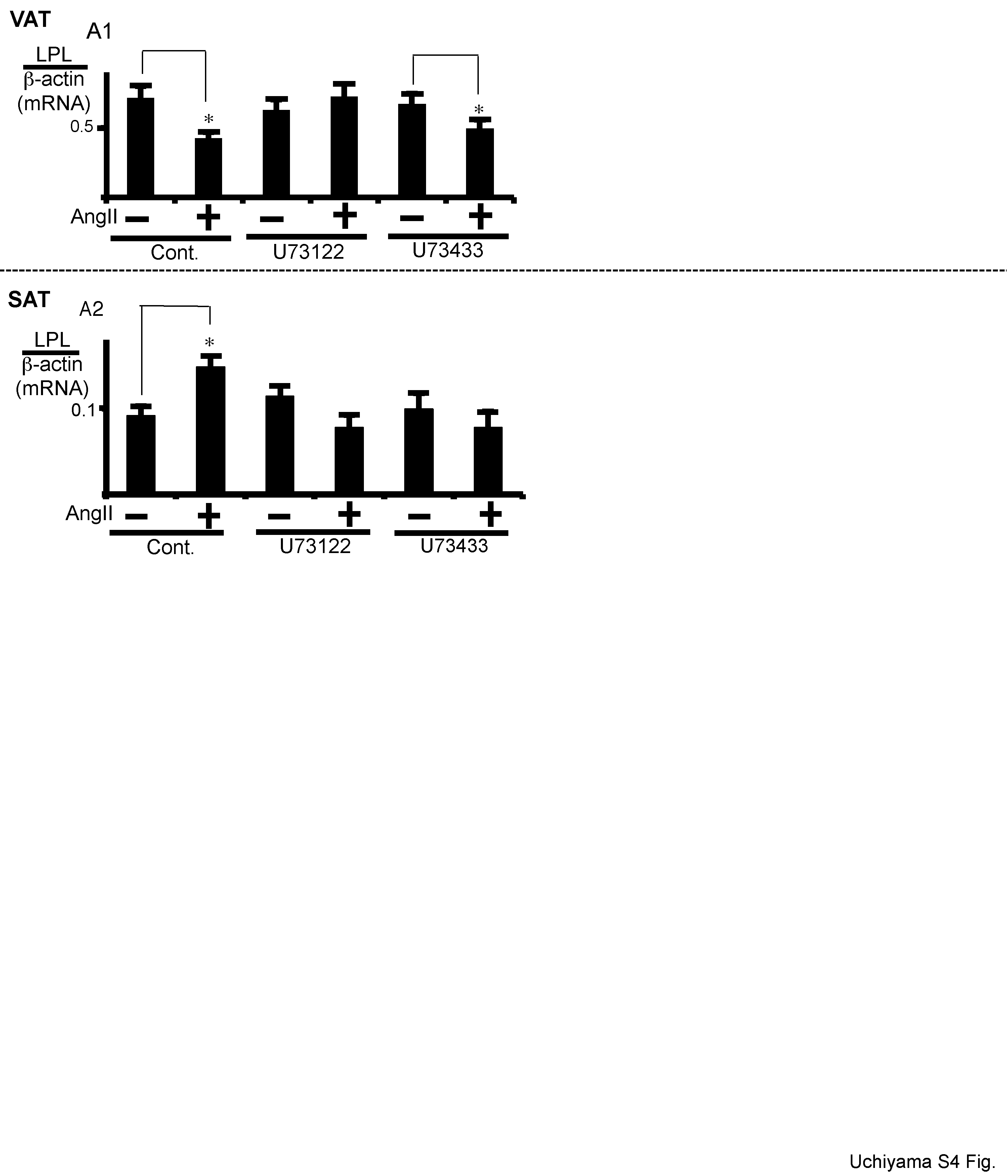

Supplement: S4 Fig — VAT or SAT were treated with U73122 (10 μM), U73343 (10 μM), or vehicle (Cont) for 1 h prior to AngII (1 μM) addition, and further incubated for 24 h to measure LPL mRNA. Each column and bar represents the mean ± SEM for three separate experiments. An asterisk (*) indicates p<0.05 vs. without AngII. LPL mRNA levels were normalized to β-actin. (TIFF) [file pone.0139638.s004.tiff]

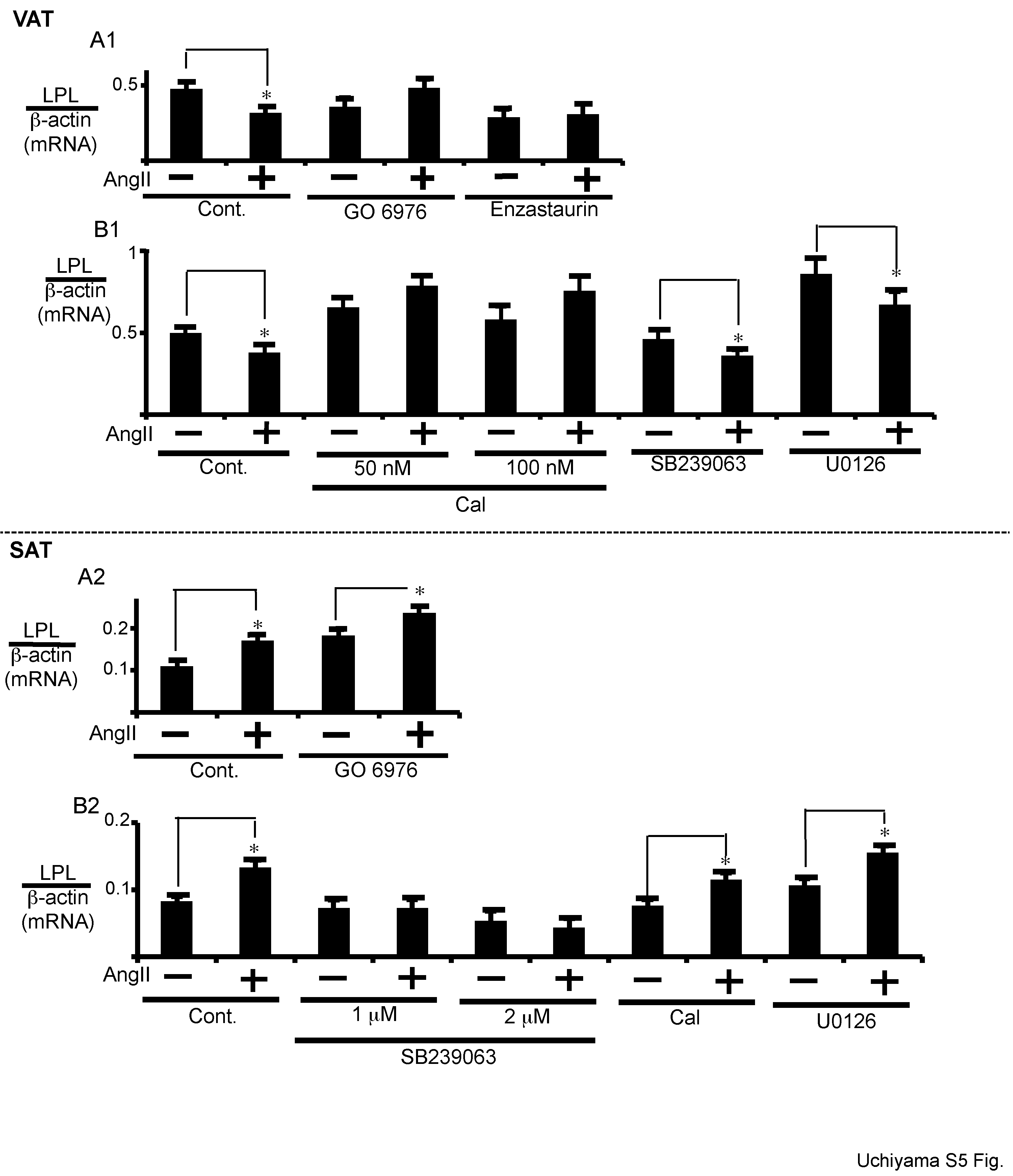

Supplement: S5 Fig — (A) VAT or SAT was treated with inhibitors of PKCβ1, i.e., 10 nM Enzastaurin (LY317615) or 10 μM GO6976, for 1 h prior to AngII (1 μM) addition, and further incubated for 24 h to measure LPL mRNA expression. (B) VAT or SAT was treated with 50 or 100 nM calphostin C (Cal), 1 or 2 μM SB239063, or 10 μM U0126 for 1 h prior to angiotensin II (1 μM) addition, and further incubated for 24 h to measure LPL mRNA expression. Each column and bar represents the mean ± SEM for three separate experiments. An asterisk (*) indicates p<0.05 vs. without AngII. The mRNA levels were normalized to β-actin. (TIFF) [file pone.0139638.s005.tiff]

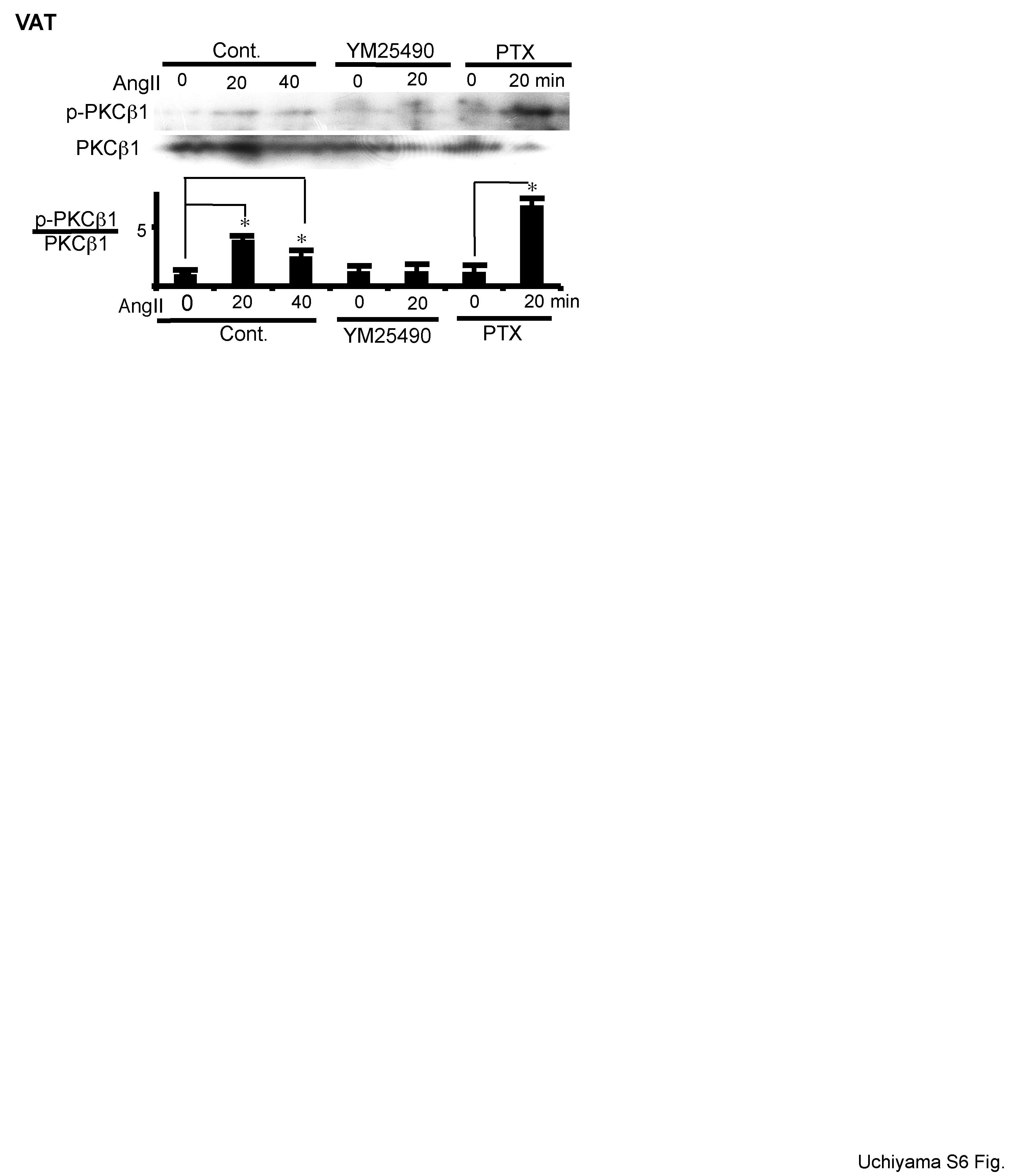

Supplement: S6 Fig — VAT was pre-treated with either PTX (100 ng/mL) or YM25490 (100 nM) for 1 h prior to AngII (1 μM) addition as described in Fig 2, and incubated for the indicated times to measure the PKCβ1 phosphorylation. The ratio of p-PKCβ1 to total PKCβ1 was calculated based on densitometric quantification of the bands. Each column and bar represents the mean ± SEM for three separate experiments performed in duplicate. An asterisk (*) indicates p<0.05 vs. without AngII. (TIFF) [file pone.0139638.s006.tiff]

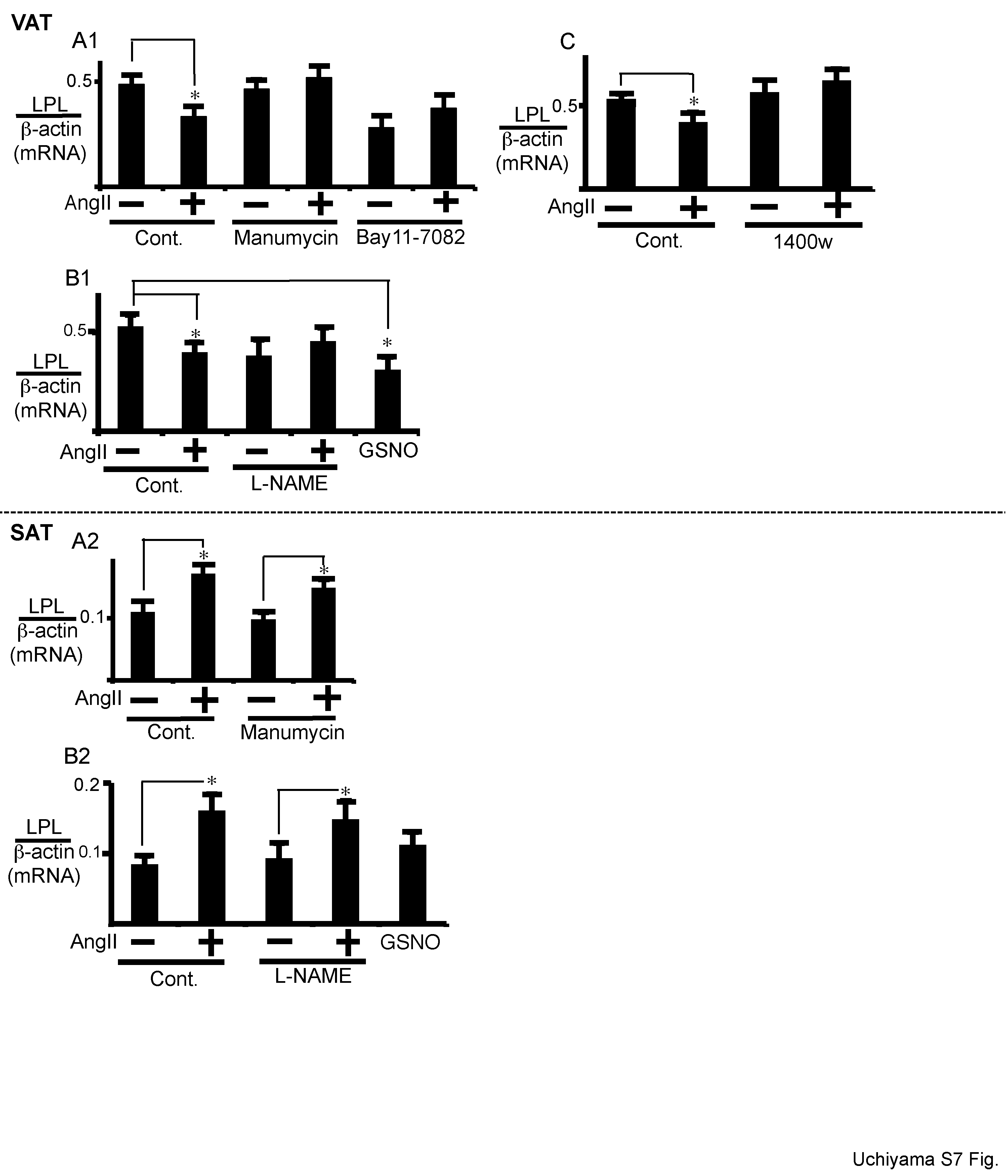

Supplement: S7 Fig — (A) Effects of IκB kinase inhibitors. VAT (A1) or SAT (A2) was pre-treated with Manumycin A (5 μM) or Bay11-7082 (5 μM) for 1 h prior to AngII (1 μM) addition and incubated for 24 h to measure LPL mRNA expression. (B and C) Effects of NOS regulators. VAT (B1 and C) or SAT (B2) was pre-treated with L-NG-nitroarginine methyl ester (L-NAME, 1 mM) or an iNOS-specific inhibitor 1400w (10 nM) for 1 h prior to AngII (1 μM) addition and incubated for 24 h to measure LPL mRNA expression. Adipose tissues were similarly incubated with S-nitro-L-glutathione (GSNO, 0.5 mM). Each column and bar represents the mean ± SEM for three separate experiments. An asterisk (*) indicates p<0.05 vs. without AngII. The mRNA levels were normalized to β-actin. (TIFF) [file pone.0139638.s007.tiff]

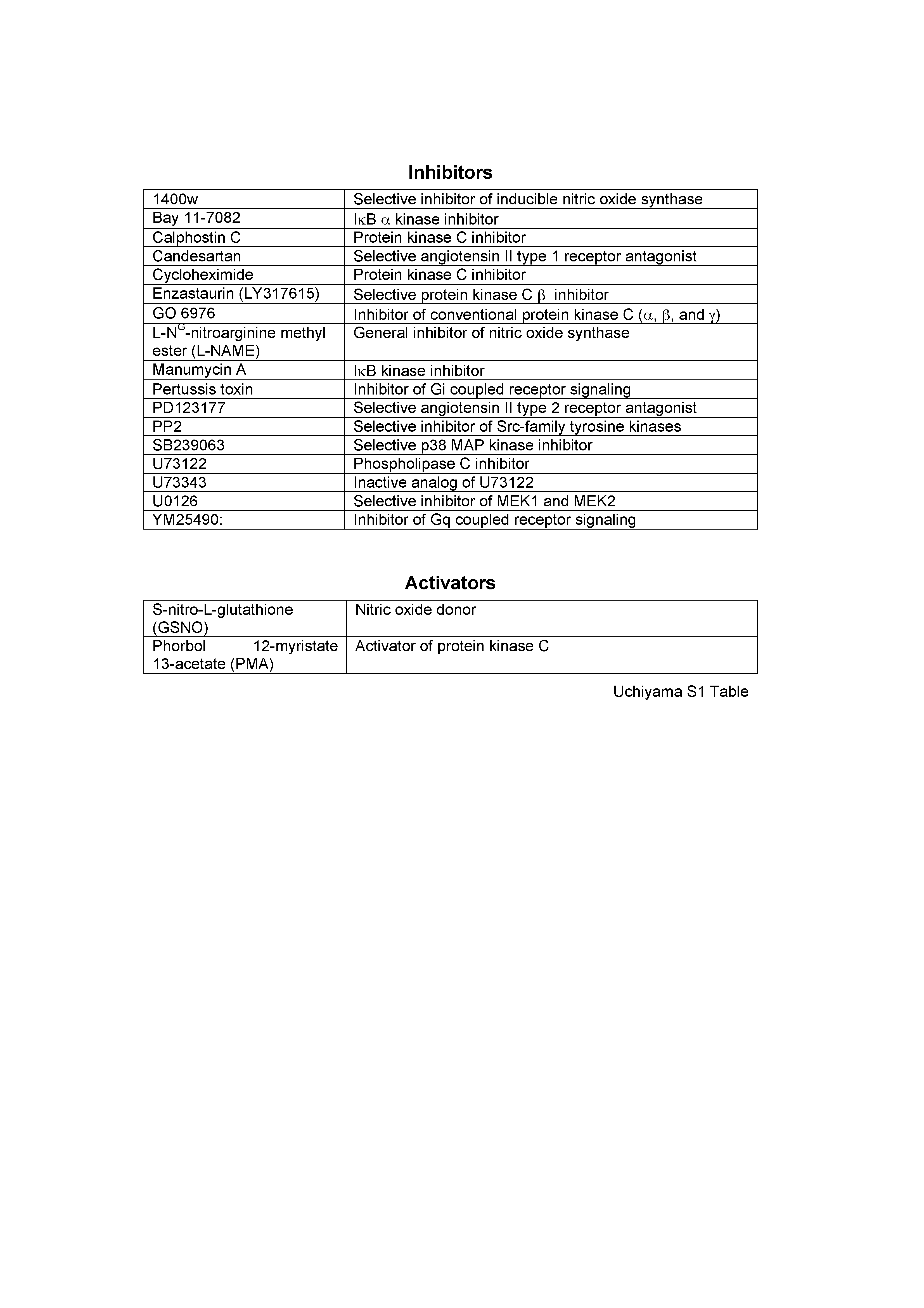

Supplement: S1 Table — (TIFF) [file pone.0139638.s008.tiff]

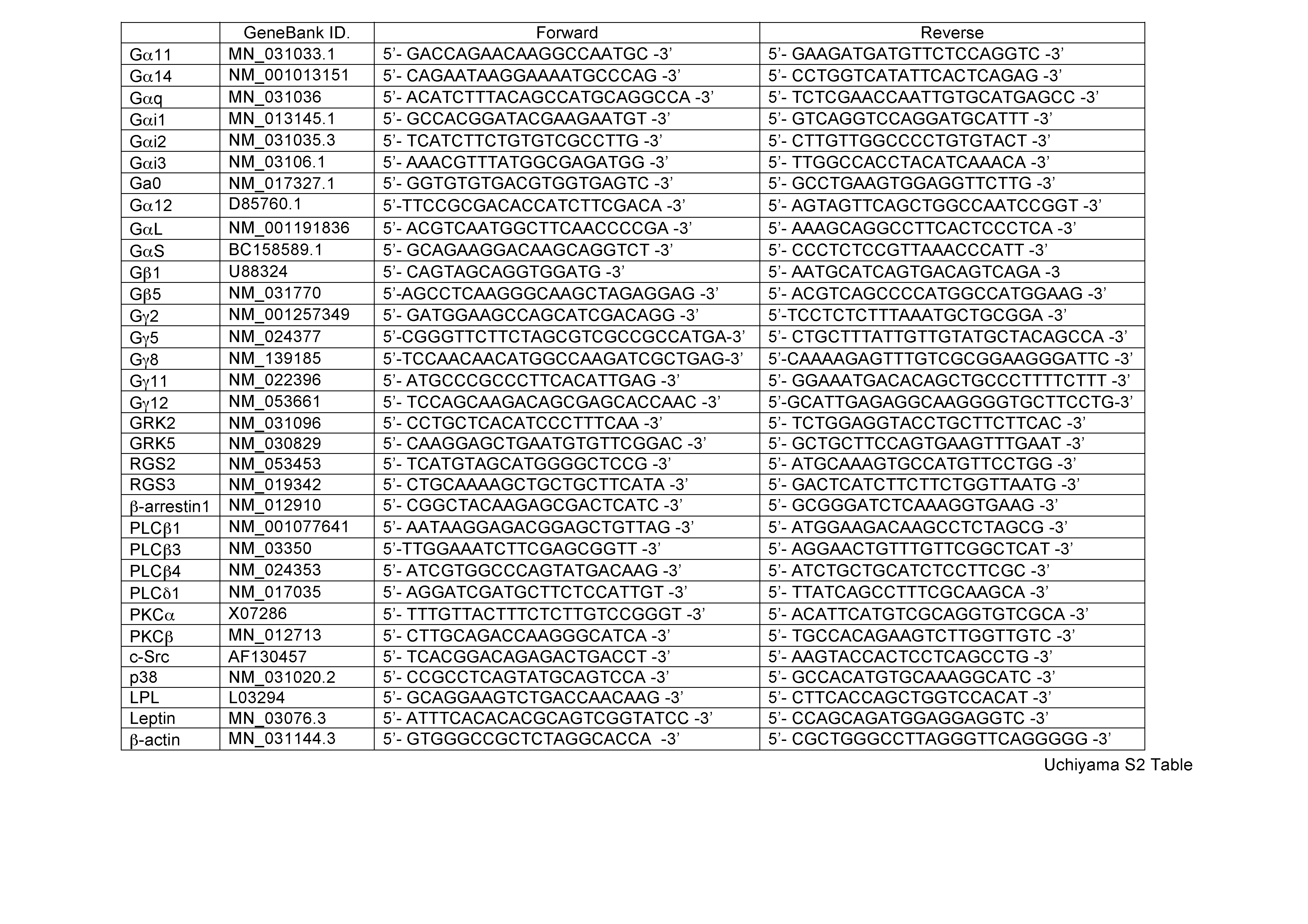

Supplement: S2 Table — GRK, G protein-coupled receptor kinase; RGS, regulator of G protein signaling; PLC, phospholipase C; PKC, protein kinase C; LPL, lipoprotein lipase; SAT, subcutaneous adipose tissue; and VAT, visceral adipose tissue. (TIFF) [file pone.0139638.s009.tiff]

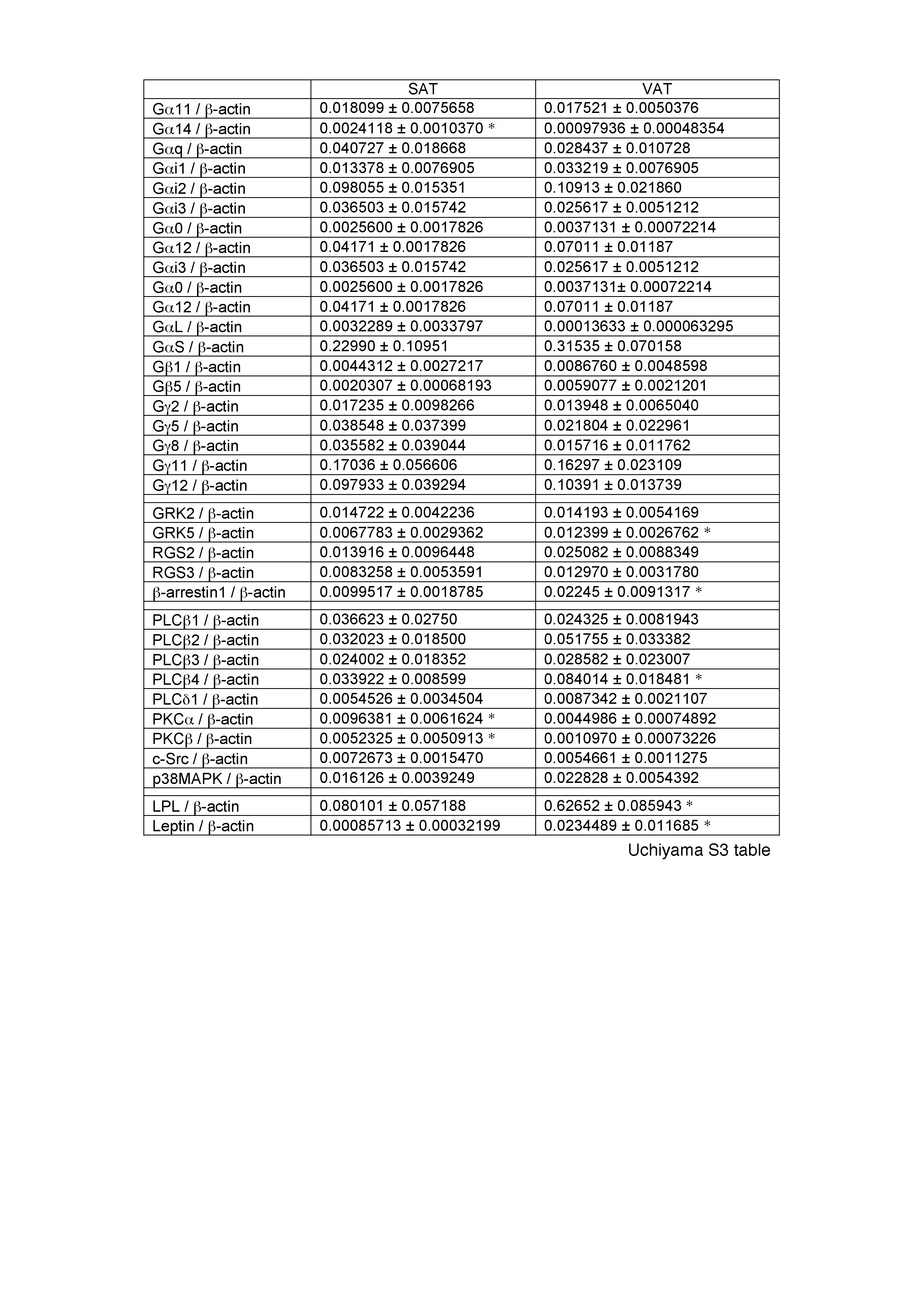

Supplement: S3 Table — Total RNA was extracted from each adipose tissue and expression of the indicated signaling proteins was analyzed using qRT-PCR. The primers used are shown in S2 Table. An asterisk (*) indicates p<0.05 vs. vehicle tissue. GRK, G protein-coupled receptor kinase; RGS, regulator of G protein signaling; PLC, phospholipase C; PKC, protein kinase C; LPL, lipoprotein lipase; SAT, subcutaneous adipose tissue; and VAT, visceral adipose tissue. (TIFF) [file pone.0139638.s010.tiff]
